# Predicting Prognosis and Immunotherapy Response in Glioblastoma (GBM) With a 5‐Gene CAF‐Risk Signature

**DOI:** 10.1002/cnr2.70158

**Published:** 2025-04-14

**Authors:** Haifeng He, Min Yan, Ke Ye, Rui Shi, Luqing Tong, Shengxiang Zhang, Yu Zhu, Renya Zhan

**Affiliations:** ^1^ Department of Neurosurgery The First Affiliated Hospital, Zhejiang University School of Medicine Hangzhou China

**Keywords:** biomarker, cancer‐associated fibroblast, glioblastoma, immunotherapy, prognosis

## Abstract

**Background:**

Cancer‐associated fibroblasts (CAF) represent significant constituents within the extracellular matrix (ECM) across a range of cancers. Nevertheless, there exists a scarcity of direct proof concerning the function of CAF in glioblastoma (GBM).

**Aims:**

This study endeavors to probe the participation of CAF in GBM by developing and validating a CAF‐risk signature and exploring its correlation with immune infiltration and immunotherapy responsiveness.

**Methods and Results:**

To fulfill these objectives, mRNA expression profiles of GBM samples and their corresponding clinical data were retrieved from two databases. First, stromal CAF‐associated genes were identified by weighted gene co‐expression network analysis (WGCNA). This method constructs co‐expression networks and pinpoints gene modules with similar expression patterns to detect relevant genes. Subsequently, a CAF‐risk signature was established via univariate and LASSO Cox regression analyses. Thereafter, gene set enrichment analysis (GSEA) and single‐sample gene set enrichment analysis (ssGSEA) were carried out to investigate the underlying molecular mechanisms. The immune status was evaluated with several R packages, and the Tumor Immune Dysfunction and Exclusion (TIDE) algorithm was utilized to assess the response to immunotherapy. Validation was performed using single‐cell RNA sequencing (scRNA) datasets, the Cancer Cell Line Encyclopedia (CCLE), and the Human Protein Atlas (HPA). Eventually, a 5‐gene (ITGA5, MMP14, FN1, COL5A1, and COL6A1) prognostic CAF model was constructed. Notably, immune infiltration analysis demonstrated a significant correlation between Treg, Macrophage, and CAF risk scores. Moreover, TIDE analysis suggested a decreased responsiveness to immunotherapy in high CAF‐risk patients. In addition, GSEA showed significant enrichment of the transforming growth factor alpha (TGF‐α), inflammatory response, and epithelial–mesenchymal transition (EMT) pathways in this subgroup. Finally, the validation through scRNA, CCLE, and HPA datasets confirmed these findings.

**Conclusion:**

The 5‐gene CAF‐risk signature exhibited accurate prognostic predictions and efficiently evaluated clinical immunotherapy responses among GBM patients. These results offer robust evidence regarding the implication of CAF in GBM and underscore the potential clinical value of personalized anti‐CAF therapies in conjunction with immunotherapy.

## 1 Introduction

Glioblastoma (GBM), constituting 70%–75% of diffuse glioma diagnoses, represents the most common and lethal primary brain tumor [1]. Annually, around 100 000 people are diagnosed with GBM worldwide [2]. As reported by Liu et al., the prognosis for GBM patients is unfavorable since the current standard‐of‐care treatments for gliomas are restricted by their toxicity to normal tissues [3]. Targeting the tumor microenvironment (TME) in GBM treatment emerges as a viable approach [4]. Simultaneously, the interaction between stromal and malignant cells plays a vital role in the development of TME in gliomas [5]. Stromal cells participate in the formation of the TME of malignant tumors [6]. Their distinctive secretory functions can adjust the acid–base balance, hypoxic state, and immunosuppressive state of the TME, and these regulatory effects can also function as therapeutic strategy targets [6, 7, 8]. Nevertheless, due to the high tumor heterogeneity, these strategies still possess certain limitations [9].

Stromal cells play a crucial role in the formation of the TME within malignant tumors. Their distinctive secretory functions are capable of modulating the acid–base balance, hypoxic state, and immunosuppressive state of the TME [7]. Nevertheless, owing to the pronounced heterogeneity of tumors, such strategies are still subject to limitations. Regrettably, to date, no target in GBM TME‐targeted therapies has demonstrated clinically significant outcomes. Cancer‐associated fibroblasts (CAF) are specialized fibroblasts present in the TME [10]. CAF interacts with another principal cell type in the TME, namely immune cells. Despite their ability to support cancer cell growth via mechanisms involving extracellular matrix (ECM) deposition and autophagy [11, 12], activated fibroblasts then aggressively remodel the matrix, release proinflammatory cytokines, and proliferate quickly during tumor development [11, 12]. Consequently, the TME becomes inflamed and immunosuppressive, thereby restricting adaptive CD8+ responses. The relative abundance of CAF can significantly affect immune infiltration across tumor locations [13]. Thus, CAF are considered a novel and crucial focus for cancer immunotherapy. However, direct evidence of CAF in GBMs is currently absent from the literature.

However, recent studies have indicated the potential presence of CAF in GBMs. For instance, recent research has identified CAF‐rich stroma in brain tumors, thereby validating the feasibility of targeting CAF in GBM treatment [13]. In addition to being a recognized oncogenic factor in GBM, fibroblast activation protein (FAP), a canonical CAF marker, was essential for healthy neural development and stem cell function [14]. According to the research by Duperret et al., elevated FAP expression correlates with high mortality in GBM [15]. Inhibition of FAP has been proposed as a novel strategy to enhance fibroblast growth factor (FGF) activity and has been successfully validated in animal models [16, 17]. CAF secretes FGFs in numerous tumors [18]. FGF2, a recognized oncogenic factor in GBM, promotes glioma development, vascularization, and self‐renewal of GBM CSCs [19]. Anti‐FGF therapy has demonstrated anti‐proliferative and anti‐angiogenic effects in glioma cell lines [20, 21]. Collectively, these lines of evidence imply that CAF signatures could potentially serve as new targets for GBM therapy. Hence, we conducted a study to explore the role of CAF signatures in GBM and to provide novel evidence corroborating the occurrence of CAF in GBM.

In this study, for the first time, we concurrently carried out a weighted gene co‐expression network analysis (WGCNA) by employing transcriptome datasets obtained from the Chinese Glioma Genome Atlas (CGGA) and The Cancer Genome Atlas (TCGA). Subsequently, hub modules exhibiting the strongest correlation with stromal CAF infiltration were identified. Thereafter, ITGA5, MMP14, FN1, COL5A1, and COL6A1 were determined as prognostic biomarkers for CAF. Utilizing univariate and least absolute shrinkage and selection operator (LASSO) Cox regression models, a 5‐gene CAF signature was established. This signature manifested the capacity to predict both glioblastoma (GBM) prognosis and therapeutic responses. CIBERSORT was deployed to evaluate the immune landscape within the entire CAF‐related risk group as well as after clustering. For the sake of validation, we made use of the single‐cell dataset GSE‐141383, the Cancer Cell Line Encyclopedia (CCLE), and the Human Protein Atlas (HPA) databases. Our results emphasize the crucial role of CAF signatures in GBM, highlighting the potential of anti‐CAF therapeutics as a novel and essential aspect in GBM treatment.

## 2 Material and Methods

### 2.1 Study Setting and Data Collection

STAR‐count data obtained from RNA sequencing analysis, combined with prognostic information (with follow‐up durations exceeding 30 days), and were collected for 593 glioblastoma (GBM) samples from The Cancer Genome Atlas (TCGA‐GBM). These data were normalized to fragments per kilobase of transcript per million mapped reads (FPKM) format using Perl version 5.30.0. Subsequently, the normalized FPKM values were converted to transcripts per million (TPM) and log‐transformed using the log2(TPM + 1) method. Additionally, we acquired normalized expression data and clinical details from 693 GBM samples in CGGA‐PRJCA001747 from the CGGA database. In cases where one gene corresponded to multiple probes, the highest value was retained. The clinical information of TCGA‐GBM and CGGA‐PRJCA001747 samples includes age, gender, survival time (year), survival status (1 = dead, 0 = alive), isocitrate dehydrogenase (IDH) status, WHO grade, and 1p/19q codeletion, which were collected from TCGA‐GBM and CGGA‐PRJCA001747 datasets. Clinical characteristic analysis was carried out by means of univariate, multivariate, and chi‐square tests. Univariate analysis was adopted to preliminarily sift through the clinical factors potentially correlated with survival outcomes, thereby facilitating the identification of a set of variables that might influence prognosis. Subsequently, multivariate analysis was employed to further dissect and ascertain the independent prognostic factors from those initially screened via univariate analysis. This step is of great significance as it enables a more precise evaluation of the genuine impact of each variable by taking into account the confounding effects of other related factors. In contrast, the chi‐square test was utilized to compare the disparities in categorical variables among different groups. For example, it could be used to compare the distribution of gender, disease subtypes, or treatment modalities among distinct patient cohorts, thereby providing valuable insights into the relationships and disparities between these categorical characteristics and the overall clinical picture. Missing values were filtered out before analysis. The risk score has been calculated based on overall survivals (OS). In the high‐risk group, OS is higher than the median OS, while in the low‐risk group, the OS is lower than the median OS.

### 2.2 Evaluation of CAF Infiltration and Computation of the Stromal Score

The abundances of CAF were assessed through the application of four disparate methodologies. First, the Estimate the Proportion of Immune and Cancer cells (EPIC) algorithm, which operates by means of cell‐type deconvolution with constrained least squares optimization. Second, the xCell algorithm functions based on gene signature enrichment. Third, the microenvironment cell population counter (MCP‐counter) exploits gene marker expression. And finally, the Tumor Immune Dysfunction and Exclusion (TIDE) algorithm. The EPIC algorithm employs cell‐type deconvolution along with constrained least squares optimization to estimate the proportions of immune and cancer cells. The xCell algorithm infers cell composition based on gene signature enrichment. The MCP‐counter calculates the number of cell populations by exploiting gene marker expression. The TIDE algorithm focuses on predicting the response of tumors to immune checkpoint blockade therapy. These algorithms analyze the infiltration of CAF from different perspectives and are of great significance in the research on the tumor microenvironment. They contribute to a deeper understanding of the roles of CAF in multiple aspects such as tumor initiation and progression, immune response, and the prediction of the efficacy of immunotherapy, providing crucial technical means and data support for tumor research and the formulation of personalized treatment regimens. The first three methodologies were implemented using the immune deconv R package (version 2.0.3), while the TIDE analysis was performed through the online platform (http://tide.dfci.harvard.edu). Additionally, the Estimation of Stromal and Immune cells in Malignant Tumor tissues using the Expression data (ESTIMATE) algorithm was applied via the estimate R package (version 1.0.13) to calculate the stromal score, indicating the extent of stromal infiltration in each tumor sample. The ESTIMATE algorithm calculates the stromal score and immune score by leveraging gene expression data from malignant tumor tissues and identifying relevant gene features based on the specific gene expression patterns of stromal cells and immune cells. Its procedures mainly revolve around the analysis of gene expression profiles. This algorithm is of great significance in the field of tumor research; it serves as an important tool for evaluating the composition of the tumor microenvironment and can reflect the heterogeneity of the tumor microenvironment. Moreover, in clinical applications, the scores generated by this algorithm can be associated with patient prognosis, providing valuable references for predicting prognosis and formulating personalized treatment strategies (such as immunotherapy).

### 2.3 Development of Coexpression Networks for CAF

Using the WGCNA R package (version 1.68), coexpression networks and hub genes associated with CAF infiltration and stromal scores were constructed and identified [22]. We began by selecting genes with the top 5000 median absolute deviations (MADs) as input for network construction in both the TCGA‐GBM and CGGA‐PRJCA001747 cohorts. Pearson's correlation similarity matrices were computed between gene pairs and adjusted to a soft‐thresholding power *β* based on scale‐free topology network criteria. The adjustment of the soft threshold power *β* based on the scale‐free topology network criteria is of great significance. Biological gene networks generally exhibit a scale‐free topology structure. An appropriate *β* value can filter out noise and false correlations, enhancing the reliability and biological relevance of the network. In terms of subsequent analysis, a proper *β* value enables the precise identification and clustering of gene modules. It is crucial for predicting the role of genes in traits or disease susceptibilities. The adjacency matrices were then clustered using the topological overlap measure (TOM), and a dynamic tree‐cut algorithm was employed to identify gene modules, each containing a minimum of 30 genes. Module eigengenes (MEs) were calculated as the first principal component of each module's expression profile, and their correlations with EPIC‐quantified CAF infiltration and stromal scores were evaluated. The module with the highest correlation was selected for further analysis. Gene significance (GS) for traits and module membership (MM) of individual genes within the identified hub module were assessed, with hub genes filtered based on stringent criteria (GS > 0.4 and MM > 0.8). Ultimately, the intersecting hub genes from both the TCGA‐GBM and CGGA‐PRJCA001747 cohorts were identified and designated as the final hub genes.

### 2.4 Analyses Using GO and KEGG

Enrichment analyses for Gene Ontology (GO) and Kyoto Encyclopedia of Genes and Genomes (KEGG) pathways were conducted on the identified hub genes to clarify their biological functions and associated pathways, employing the ClusterProfiler R package (version 3.14.3) [23]. A significance threshold of *p* < 0.05 was utilized. Enriched genes were evaluated using a hypergeometric test to calculate the corresponding z‐score value with the GO‐plot package, utilizing provided gene values [24]. The log fold change (LogFC) values of genes were obtained from TCGA‐GBM.

### 2.5 Development and Validation of Prognostic Models

To construct the CAF risk model, the larger sample size of the CGGA‐PRJCA001747 cohort was utilized, while the TCGA‐GBM cohort, comprising 593 samples, served as the validation set. Univariate Cox regression analysis was employed to identify stromal CAF hub genes as prognostic indicators for OS. A risk score was calculated to indicate prognosis, with higher scores correlating with poorer outcomes. Genes with *p* values < 0.05 were then subjected to LASSO Cox regression analysis, performed with 1000 iterations using the glmnet R package (version 4.1‐7). The CAF risk model was established using the formula: CAF risk score = ∑ (*βi* * Expi), where *βi* represents the LASSO coefficient and Expi denotes the expression value of the *i*th gene. For example, SHC1, SERPINH1, LAMC1, NRP1, MMP14, COL5A2, LOXL2, COL6A1, COL1A2, COL1A2, and COL6A2, as the classic markers of CAF, deserve attention. Their Lasso coefficients are −0.06, 0.23, 0.29, −0.26, 0.06, 0.10, 0.08, −0.02, −0.08, −0.02, and 0.06, respectively. In TCGA‐74‐6577, the expression levels of these genes are 4.96, 6.54, 5.06, 3.91, 6.39, 4.26, 4.57, 7.95, 5.91, 5.94, and 6.96 respectively. Therefore, the CAF risk score of TCGA‐74‐6577 is: (−0.06 * 4.96) + (0.23 * 6.54) + (0.29 * 5.06) + (−0.26 * 3.91) + (0.06 * 6.39) + (0.10 * 4.26) + (0.08 * 4.57) + (−0.02 * 7.95) + (−0.08 * 5.91) + (−0.02 * 5.94) + (0.06 * 6.96) = 2.50. GBM patients were divided into high and low CAF risk groups based on their median CAF risk scores. The Kaplan–Meier curve analysis and log‐rank test were conducted to assess differences in OS between these groups. The CAF risk model's validation was carried out within the TCGA‐GBM cohort. Clinical data from both cohorts were analyzed using univariate and multivariate Cox regression, Chi‐square tests, and receiver operating characteristic (ROC) curve analysis, comparing them with OS.

### 2.6 The Acquisition and Correlation Evaluation of CAF Markers

CAF‐specific and nonspecific markers were sourced from prior research. To assess the robustness of our CAF model markers in GBM, we conducted Spearman's correlation analyses between the CAF risk score and stromal score, in addition to various CAF infiltration estimations (EPIC, xCell, MCP‐counter, and TIDE). Additionally, the correlations between CAF model genes and established CAF markers were examined in the TCGA‐GBM and CGGA‐PRJCA001747 cohorts.

### 2.7 Predicting Responses to Chemotherapy and Immunotherapy

The Genomics of Drug Sensitivity in Cancer (GDSC) database was utilized to estimate IC50 values for commonly used drugs in GBM samples, including bleomycin, lapatinib, paclitaxel, camptothecin, cisplatin, docetaxel, methotrexate, and sunitinib. This estimation was performed using ridge regression with tenfold cross‐validation via the pRRophetic R package (version 0.5). Additionally, the TIDE online algorithm (http://tide.dfci.harvard.edu/) was employed to predict responses to immune checkpoint blockade therapy. Objective response rates and survival data for the TCGA‐GBM cohort were obtained using the IMvigor210CoreBiologies R package (http://research‐pub.gene.com/IMvigor210CoreBiologies/). Responses to immunotherapy—categorized as complete response (CR), partial response (PR), stable disease (SD), and progressive disease (PD)—were compared between low‐ and high‐risk CAF groups. Differences in response rates were evaluated using the chi‐square test, and the predictive performance of the CAF risk signature was assessed through receiver operating characteristic (ROC) curve analysis and area under the curve (AUC) calculations.

### 2.8 Collection and Analysis of Somatic Alteration Data

TCGA‐GBM cohort somatic mutation data were processed using Perl v5.30.0. The top 20 mutations with the highest frequencies in both low and high CAF risk groups were identified and visualized utilizing the maftools R package (version 2.14.0) in conjunction with Perl v5.30.0. Utilizing Perl, we conducted screening and extraction of variant genes from the MAF files retrieved from TCGA‐GBM. Subsequently, data cleansing and preprocessing were performed with Perl. Upon inputting the CAR‐Risk genes, we were able to align the somatic mutation data with them and categorize the mutation data of each sample into the corresponding risk groups, thus obtaining the Maf files for the high‐risk and low‐risk groups. Thereafter, the mutation frequency was tallied and sorted using the maftools::oncoplot function within the maftools package of R, and the outcomes were visualized (Figure S5A,B). TMB, recognized as a potential predictor of immunotherapy efficacy, was calculated for each GBM sample via the tmb() function within the maftools package. Spearman's correlation analysis assessed the relationship between TMB and CAF risk scores. The proportion of immune and stromal cells in the TME was estimated using the ESTIMATE method (https://sourceforge.net/projects/estimateproject/).

### 2.9 Assessment of Immune Status

Immune checkpoint analysis in R was utilized to elucidate CAF's role in the GBM immune microenvironment. The CIBERSORT algorithm (https://cibersort.stanford.edu/) was employed to quantify the relative abundance of tumor‐infiltrating immune cells in each GBM sample. The study focused on key immune checkpoints, including ATIC (5‐aminoimidazole‐4‐carboxamide ribonucleotide formyltransferase), OLA1 (Obg‐like ATPase‐1), CTLA4 (cytotoxic T‐lymphocyte‐associated protein‐4), PDCD1 (Programmed cell death‐1), PDCD1LG2 (Programmed cell death‐1 ligand‐2), CD274, IDO1 (Indoleamine 2,3‐Dioxygenase‐1), and HAVCR2 (T cell immunoglobulin and mucin domain‐3). Comprehensive immune status analysis was conducted using a suite of R packages: TIMER (version 2.0), CIBERSORT (version 1.03), CIBERSORT‐ABS (version 1.03), QUANTISEQ (version 4.10), and deconv (version 3.17), which incorporated MCPcounter, Xcell, and EPIC, all within R v4.1.1.

### 2.10 Analysis of GSEA and ssGSEA

Gene Set Enrichment Analysis (GSEA) was utilized to identify enriched hallmark and immune gene sets in high and low CAF risk groups within the TCGA‐GBM and CGGA‐PRJCA001747 datasets. This analysis employed the enrichplot (version 1.18.4) and clusterProfiler R packages. Gene sets “h.all.v7.4.symbols” and “immune.gmt” were sourced from the Molecular Signatures Database (MSigDB). Additionally, single‐sample GSEA (ssGSEA) was used to calculate enrichment scores for TNF‐α, inflammatory, and epithelial–mesenchymal transition (EMT) hallmark gene sets. Spearman's correlation analysis was then performed to assess the relationship between CAF risk scores and gene set enrichment scores, including the enrichment of regulatory T cells (Tregs) identified by ssGSEA in both the TCGA‐GBM and CGGA‐PRJCA001747 cohorts.

### 2.11 Immunotherapy Analysis

The TIDE and microsatellite instability (MSI) expression signatures were used to evaluate potential immunotherapy efficacy in low‐ and high‐risk groups stratified by the chromatin organization‐related gene signature (CORGS). TIDE scores were computed using the TIDE website (https://tide.dfci.harvard.edu), while MSI scores were derived by averaging the log2‐scale normalized expression levels of 18 signature genes. Furthermore, Immunophenoscore (IPS) data, obtained from The Cancer Imaging Archive (TCIA) website (https://www.tcia.at/home), and IC50 values calculated via the “pRRophetic” package in R, were used to predict responses to immune checkpoint inhibitors and common targeted therapies in CAF low‐ and high‐risk groups, respectively.

### 2.12 Validation of the CAF Risk Signature Was Conducted Using scRNA‐Seq, the CCLE, and the HPA Databases

The GSE‐141383 dataset was chosen for single‐cell RNA verification of the CAF risk signature. Cell clusters and types, Kruskal test for different cell types, identification of the five hub genes, cell–cell interaction (CCI), and translation factors (TFs) analysis were conducted using the TISCH2 website (http://tisch.comp‐genomics.org). TLS, TLS‐melanoma, T cell‐inflamed, IFNG, checkpoint, IMPRES, IPRES, CTL, and T‐quiescent were selected for analysis. To validate at the cellular level, mRNA expression levels of the identified markers were obtained from 38 fibroblast and 39 GBM cell lines listed in the Cancer Cell Line Encyclopedia (CCLE) database (https://portals.broadinstitute.org/ccle). The expression patterns in fibroblast and GBM cell lines were analyzed using heatmaps and Wilcoxon tests. Additionally, for protein‐level analysis, immunohistochemical (IHC) staining images of these markers in GBM tissues were sourced from the Human Protein Atlas (HPA) online database (https://www.proteinatlas.org/), facilitating direct examination of target protein localization.

### 2.13 Statistical Analysis

Statistical analyses were conducted using R software (version 4.1.1; https://www.r‐project.org/). The median CAF risk score served as the cutoff for classifying GBM patients into high and low CAF risk groups. Pairwise comparisons were performed using the Wilcoxon and chi‐square tests. For assessing overall survival (OS), Kaplan–Meier curve analysis and the log‐rank test were employed, utilizing the survival (version 3.5‐5) and survminer (version 0.4.9) R packages. The Kruskal–Wallis test was utilized for single‐cell RNA analysis. A *p* value of < 0.05 was considered statistically significant. All P‐values resulting from multiple comparisons have been adjusted using Bonferroni adjustment.

## 3 Experimental Materials

This study does not involve any experimental materials.

## 4 Results

### 4.1 Clinical Information of Patients Was Analyzed Based on OS

Clinical characteristics associated with OS were analyzed using univariate analysis in both the TCGA‐GBM and CGGA‐PRJCA001747 cohorts. In TCGA‐GBM, age and IDH mutation status showed significant differences, while no significant difference was observed between genders (Table 1). Only WHO grade IV and non‐codel 1p19q status were available for collection in this cohort. In CGGA‐PRJCA001747, age, WHO grade, IDH mutation, and 1p19q mutation status showed meaningful differences in OS, with no significant difference observed between genders (Table 2). Multivariate analysis was conducted for further exploration using nomogram plots (Figure S1A,D), and ROC analysis was employed for 1‐, 3‐, and 5‐year predictions (Figure S1B,E) in both cohorts, with significant AUC values observed. Chi‐square tests were utilized for deeper analysis. In TCGA‐GBM, only IDH mutation status showed significant differences (Figure S1C), whereas in CGGA‐PRJCA001747, age, IDH mutation status, and 1p19q codeletion status were significantly different (Figure S1F). These results indicate that both TCGA‐GBM and CGGA‐PRJCA001747 cohorts exhibit significant clinical characteristics for GBM prognosis analysis and prediction.

**TABLE 1:** Univariate analysis of TCGA clinical characteristics analysis based on OS.

Characteristics	Total (*N*)	Overall survival (OS)	
		Hazard ratio (95% CI)	*p*
Age	160	1.026 (1.012–1.041)	< 0.001
Sex	160		0.987
Male	104	Reference	
Female	56	1.003 (0.695–1.448)	0.987
WHO grade	160		
WHO IV	160	—	—
IDH mutation	160		
WT	143	Reference	
Mutant	17	0.254 (0.110–0.585)	< 0.001
1p19q codeletion status	160		—
Non‐codel	160	—	—
Score	160	4.150 (1.747–9.854)	< 0.001

**TABLE 2:** Univariate analysis of CGGA clinical characteristics analysis based on OS.

Characteristics	Total (*N*)	Overall survival (OS)	
		Hazard ratio (95% CI)	*p*
Age	657	1.026 (1.018–1.035)	< 0.001
Sex	657		0.563
Male	374	Reference	
Female	283	0.943 (0.771–1.152)	0.563
WHO grade	657		< 0.001
WHO II	172	Reference	
WHO III	248	2.545 (1.846–3.509)	< 0.001
WHO IV	237	6.972 (5.085–9.561)	< 0.001
IDH mutation	609		< 0.001
Wildtype	276	Reference	
Mutant	333	0.323 (0.262–0.398)	< 0.001
1p19q codeletion status	591		< 0.001
Non‐codel	454	Reference	
Codel	137	0.268 (0.193–0.372)	< 0.001
Score	657	5.839 (3.958–8.614)	< 0.001

### 4.2 Elevated CAF Infiltration and Stromal Scores Are Correlated With Reduced OS in Patients With GBM

**FIGURE 1:**
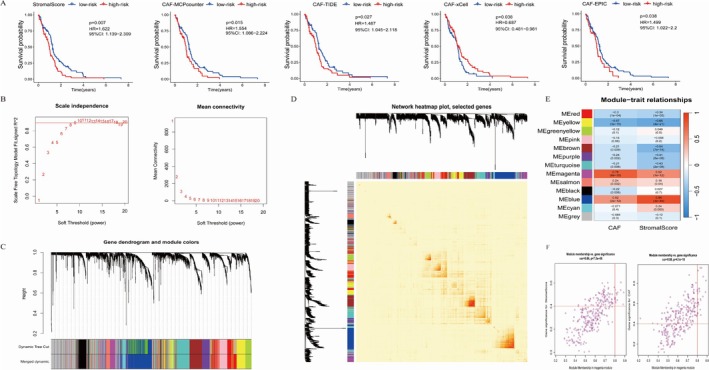
Kaplan–Meier analyses based on CAF‐related scores and the coexpression network constructed by WGCNA in the TCGA‐GBM cohort. (A) Kaplan–Meier analyses based on stromal scores, MCPcounter, TIDE, xCell, and EPIC. Note. GBM patients in the TCGA‐GBM cohort. (B) A soft‐threshold power (*β*) of 8 was chosen in the TCGA‐GBM cohort based on the scale‐free topology criterion. (C) Clustering dendrograms revealed that genes with similar expression patterns were grouped into coexpression modules in the TCGA‐GBM cohort. (D) TOM plot of cogenes. (E) Scatter plots illustrating the module membership (MM) and gene significance (GS) of each gene within the magenta module for the TCGA‐GBM cohort. (F) The correlation between the gene and the coexpression module is represented on the horizontal axis, while the vertical axis displays the correlation between the gene and the phenotype.

**FIGURE 2:**
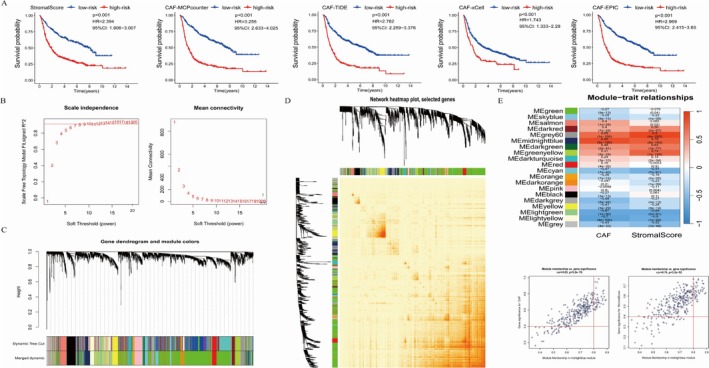
Kaplan–Meier analyses based on CAF‐related scores and the coexpression network constructed by WGCNA in the CGGA‐PRJCA001747 cohort. (A) Kaplan–Meier analyses based on stromal scores, MCPcounter, TIDE, xCell, and EPIC. Note. GBM patients in the CGGA‐PRJCA001747 cohort. (B) The soft‐threshold power (*β*) of 8 was chosen according to the scale‐free topology criterion within the CGGA‐PRJCA001747 cohort. (C) Clustering dendrograms indicated that genes exhibiting similar expression patterns were grouped into coexpression modules within the CGGA‐PRJCA001747 cohort. (D) TOM plot of cogenes. (E) Scatter plots illustrating the module membership (MM) and gene significance (GS) for each gene within the magenta module in the CGGA‐PRJCA001747 cohort; The relationship between the gene and the coexpression module is represented, with the vertical axis depicting the correlation between the gene and the phenotype.

Upon comprehension of the clinical traits of patients, a deeper exploration into the infiltration of CAF within GBM and its correlation with prognosis was conducted. Multiple techniques such as EPIC, xCell, MCP‐counter, and TIDE were employed to analyze CAF infiltration, and stromal scores were obtained through the ESTIMATE algorithm. The prognostic significance of CAF infiltration and stromal scores concerning OS was examined with the log‐rank test. Kaplan–Meier curves revealed a significant correlation between heightened CAF infiltration and stromal scores and reduced OS in GBM patients from both the TCGA‐GBM and CGGA‐PRJCA001747 cohorts (Figures 1A and 2A). This underscores the need for additional research on CAF and stromal‐related genes in GBM. The CAF abundances and stromal scores obtained from EPIC were used for subsequent analyses, while data from the other three methods were employed for external validation of the identified CAF model.

### 4.3 Coexpression Network of CAF and Stromal Scores

WGCNA was performed on the TCGA‐GBM and CGGA‐PRJCA001747 cohorts. A soft threshold power (*β*) of 8 was determined for constructing a scale‐free topology network in both cohorts. The *β* value is a crucial parameter for transforming the correlation coefficient matrix into an adjacency matrix. As shown in Figures 1B and 2B, we observed the topological properties of the network under different *β* values through the scale‐free topology fit index. When the soft Threshold in the Scale‐Free Topology Criterion was set to 8, it was closest to the optimal fitting value of the scale‐free topology fit index in both the TCGA and CGGA cohorts. Therefore, it is reasonable to choose 8 as the *β* value. In TCGA‐GBM and CGGA‐PRJCA001747, hierarchical clustering trees revealed 13 and 20 co‐expression modules, respectively. In the TCGA‐GBM cohort (Figure 1C,D), the magenta module showed the highest positive correlation with CAF proportion and stromal score. In the CGGA‐PRJCA001747 cohort, the midnight blue module exhibited the strongest positive correlation (Figure 2C,D). Using threshold criteria of MM > 0.8 and GS > 0.4, 24 hub genes in the black module of TCGA‐GBM and 41 hub genes in the brown module of CGGA‐PRJCA001747 were identified as highly correlated with CAF and stromal scores (Figures 1E,F and 2E).

### 4.4 Functional Analyses

**FIGURE 3:**
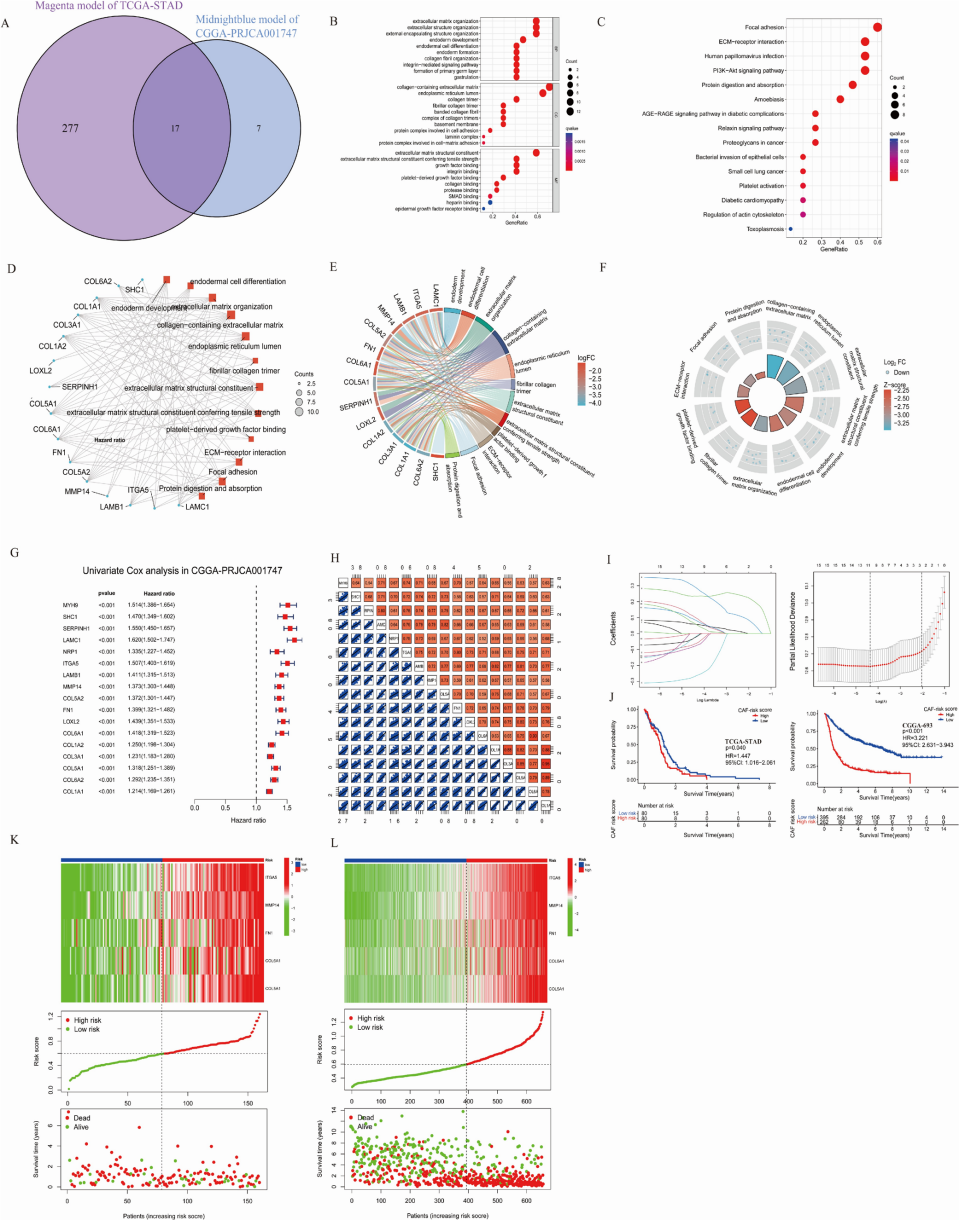
GO/KEGG enrichment of 17 hub genes and the 5‐gene risk model in both the TCGA‐GBM and CGGA‐PRJCA001747 cohorts. Note. (A) The Venn diagram illustrates the overlap between magenta module genes from the TCGA‐GBM dataset and midnight blue module genes from the CGGA‐PRJCA001747 dataset. (B) GO analysis reveals enriched terms for biological processes (BP), cellular components (CC), and molecular functions (MF). (C, D) KEGG pathway analysis of the 17 genes is depicted through a bar plot and network visualization. (E, F) KEGG analysis incorporating the log fold change (logFC) of these 17 genes is shown for the TCGA‐GBM cohort. (G) Univariate Cox regression analysis was performed to identify genes associated with overall survival in the CGGA‐PRJCA001747 cohort. (H) A correlation heatmap of hub genes in the CGGA‐PRJCA001747 cohort was generated. (I) The least absolute shrinkage and selection operator (LASSO) Cox regression analysis produced coefficient profiles, and the optimal lambda parameter was determined using tenfold cross‐validation to formulate the risk model. (J) Kaplan–Meier survival analysis indicated that high‐risk GBM patients exhibited reduced overall survival in both the TCGA‐GBM and CGGA‐PRJCA001747 cohorts. (K, L) The risk score distribution, survival status, and expression heatmaps of ITGA5, MMP14, FN1, COL6A1, and COL5A1 were analyzed for the TCGA‐GBM and CGGA‐PRJCA001747 cohorts.

Analyzing the intersection of two hub gene sets revealed 17 common genes, including MYH9, SHC1, SERPINH1, LAMC1, NRP1, ITGA5, LAMB1, MMP14, COL5A2, FN1, LOXL2, COL6A1, COL1A2, COL3A1, COL5A1, COL6A2, and COL1A1, as depicted in Figure 3A. GO and KEGG analyses were performed on these 17 genes, elucidating their functional roles (Figure 3B–D). Functional pathway enrichment was qualitatively assessed based on the log fold change (logFC) of these genes in the TCGA‐GBM dataset (Figure 3E,F), revealing enrichment in pathways related to ECM organization, focal adhesion, and signaling pathways such as PI3K‐Akt.

### 4.5 Construction of the Stromal CAF‐Based Prognostic Risk Model

The research employed a training cohort consisting of 693 GBM samples from CGGA‐PRJCA001747 and a validation cohort of 160 samples from TCGA‐GBM, chosen for their larger sample size. Univariate Cox regression analysis of 17 common hub genes indicated that all were significantly linked to OS, with *p* values < 0.05 (Figure 3G). Coexpression assessments confirmed significant correlations between these 17 hub genes and OS (Figure 3J). Following this, LASSO Cox regression analysis was conducted (Figure 3H), resulting in the selection of five genes—COL5A1, COL6A1, FN1, ITGA5, and MMP14—to construct the CAF‐risk model. Using the median risk score as a cutoff, GBM patients were stratified into high and low CAF‐risk groups. Kaplan–Meier curves illustrated that patients in the high CAF‐risk group had significantly poorer OS compared to those in the low CAF‐risk group across both the TCGA‐GBM and CGGA‐PRJCA001747 cohorts (Figure 3I). Figure 3K depicts the relationship between the five genes and samples in TCGA, while Figure 3L shows their association with risk scores. These results highlight the critical role of CAF and stromal signature genes as key prognostic indicators in GBM.

### 4.6 CAF Signature Correlated With Both CAF Infiltration and the Expression of CAF Markers

**FIGURE 4:**
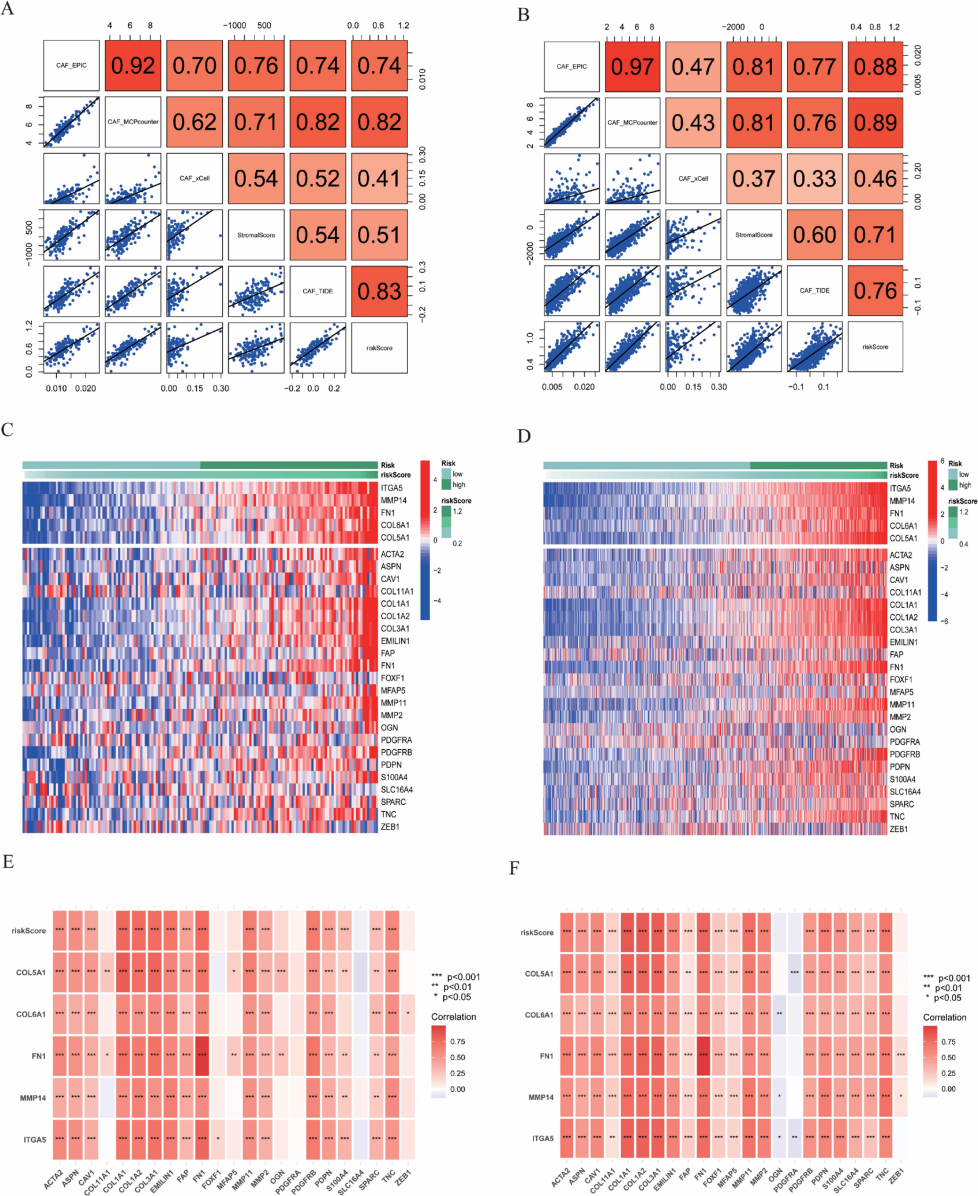
Spearman's correlation analyses demonstrated a robust positive association between the CAF risk score and both stromal scores and various measures of CAF infiltration in the TCGA‐GBM (A) and CGGA‐PRJCA001747 (B) cohorts. The heatmap illustrated the expression profiles of CAF markers, highlighting four CAF genes correlated with the CAF risk score in the TCGA‐GBM (C) and CGGA‐PRJCA001747 (D) cohorts. Furthermore, the CAF risk score and five signature genes showed a positive correlation with previously reported CAF markers in the literature for the TCGA‐GBM (E) and CGGA‐PRJCA001747 (F) cohorts. *p < 0.05, **p < 0.01, ***p < 0.001.

We performed Spearman's correlation analyses to assess the predictive accuracy of the CAF model concerning CAF infiltration. The CAF risk score exhibited robust positive correlations with CAF abundances estimated by EPIC, xCell, MCP‐counter, and TIDE, in addition to the stromal score in both the TCGA‐GBM (Figure 4A) and CGGA‐PRJCA001747 cohorts (Figure 4B). Moreover, a strong positive correlation was identified between the CAF risk score and the expression levels of five genes associated with various CAF markers in both the TCGA‐GBM (Figure 4C,E) and CGGA‐PRJCA001747 cohorts (Figure 4D,F). Consequently, five CAF‐related genes were selected for the construction of the prognostic risk model. Notably, each gene demonstrated a significantly lower OS in the high CAF risk group across both the TCGA (Figure S2A, above) and CGGA cohorts (Figure S2A, below).

### 4.7 Sensitivity of CAF Risk Groups to Chemotherapeutic Agents

The IC50 values for various drugs were extracted from the GDSC database. Wilcoxon analyses identified significant differences in IC50 values between high and low CAF risk groups among GBM patients. High CAF‐risk GBM patients demonstrated increased sensitivity to erlotinib and AZD3759, whereas low CAF‐risk GBM patients exhibited greater sensitivity to AZ690, dasatinib, SCH772984, OTX015, and NU7441. These findings were consistent across both the TCGA‐GBM (Figure S2B) and CGGA‐PRJCA001747 (Figure S2C) cohorts.

### 4.8 GSEA of the Five‐Gene CAF Signature

To explore the functional enrichment of the CAF signature in the high‐risk group, we conducted GSEA on the TCGA‐GBM and CGGA‐PRJCA001747 datasets among individuals with high CAF risk. Significant enrichment was observed in hallmark signaling pathways, particularly TNF‐α, inflammatory response, and EMT pathways, across both cohorts (Figure S3A,B). The ssGSEA results indicated a positive correlation between the CAF risk score and enrichment scores for TNF‐α, inflammatory response, and EMT in both the TCGA‐GBM (Figure S3C) and CGGA‐PRJCA001747 cohorts (Figure S3C). TNF‐α, EMT induction, and inflammatory responses are highly correlated with promoting the invasiveness and metastatic potential of tumor cells as well as modifying the tumor immune microenvironment. It has been evidenced that TNF‐α augments the invasion and metastasis of tumor cells by activating the NF‐κB signaling pathway and inducing EMT [25, 26]. Additionally, the role of TNF‐α as a pro‐inflammatory cytokine is inextricably linked with the inflammatory response. These findings imply that anti‐inflammatory strategies might also represent one of the possible avenues for improving the prognosis of GBM.

The outcomes of GSEA and ssGSEA indicate the significance of immunity in the high CAF‐risk group. Further, GSEA and ssGSEA focusing on immune function revealed enrichment of CCRs, TILs, and Tregs in both the TCGA‐GBM (Figure S4A) and CGGA‐PRJCA001747 cohorts (Figure S4B). In ssGSEA, Tregs showed a significant association with the CAF risk score in both the TCGA‐GBM (Figure S4C) and CGGA‐PRJCA001747 cohorts (Figure S4D). In ssGSEA, Tregs were significantly correlated with the CAF risk score in both TCGA‐GBM and CGGA‐PRJCA001747 cohorts. This underscores the significant role of immunity in the GBM tumor microenvironment influenced by CAF, with Tregs likely playing a pivotal role in its immune function.

### 4.9 Correlation Between CAF Signatures and Somatic Mutations

We identified and visualized the top 20 genes with the highest mutation frequencies in both high‐ and low‐risk CAF subgroups using waterfall plots. Several genes were commonly mutated across both risk groups, including PTEN, TP53, EGFR, TTN, MUC16, FLG, RYR2, NF1, PIK3CA, ATRX, LRP2, PIK3R1, RB1, SPTA1, LRP1, PCLO, MUC17, IDH1, HMCN1, and DNAH5. Notably, mutations in PTEN (38%), TP53 (28%), EGFR (26%), TTN (31%), and NF1 (15%) were significantly enriched in the high‐risk CAF group (Figure S5A). Within this group, the most frequently mutated genes were TP53 (38%), EGFR (28%), PTEN (26%), TTN (26%), and MUC16 (22%) (Figure S5B).

Additionally, we observed significant correlations between TMB values and OS in TCGA‐GBM samples (Figure S5C). An integrated analysis incorporating the CAF risk score revealed notable differences in OS and TMB values (Figure S5D). These results highlight the influence of CAF‐related gene mutations on the prognostic outcomes for patients with GBM.

### 4.10 TME Immune Situation Analysis Based on CAF Risk Groups in TCGA‐GBM

**FIGURE 5:**
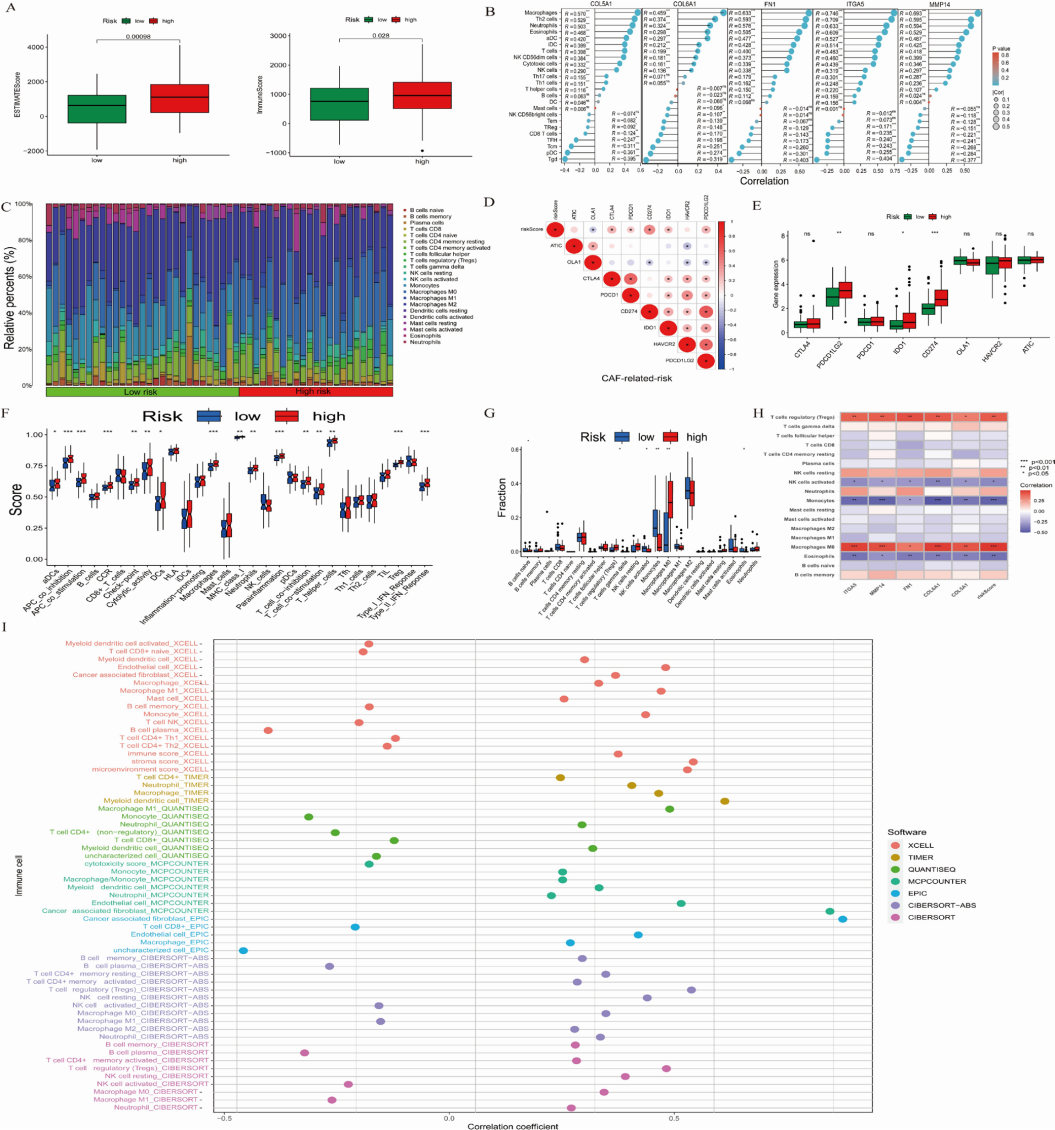
A thorough examination of the immune environment in relation to CAF risk groups within the TCGA‐GBM cohort. (A) Variations in ESTIMATE and immune scores between CAF risk groups. (B) Correlation between known immune checkpoints and CAF risk scores. (C) Differences in stromal scores across CAF risk groups. (D) Immune cell infiltration associated with five specific genes. (E) Differences in known immune checkpoints between CAF risk groups. (F) Relative proportions of TME immune cell types. (G) Variations in TME immune cell types by CAF risk groups. (H) Correlation between TME immune cell types and CAF risk groups. (I) Differences in immune function within the GBM TME relative to CAF risk groups. Correlation among different immune estimation methods (TIMER, CIBERSORT, CIBERSORT‐ABS, QUANTISEQ, MCPcounter, Xcell, and EPIC) in the TCGA‐GBM dataset.

In the TCGA‐GBM cohort, we conducted CIBERSORT and ESTIMATE analyses to enhance our understanding of immune function. Our ESTIMATE analysis revealed significant differences in the ESTIMATE score (Figure 5A, left) and immune score (Figure 5A, right) between high‐ and low‐risk CAF groups. Subsequently, we analyzed immune cell infiltration for each gene (Figure 5B) and evaluated the distribution of various immune cell types across the CAF low‐ and high‐risk groups (Figure 5C). We also examined correlations with established immune checkpoints (ATIC, OLA1, CTLA4, PDCD1, CD274, IDO1, HAVCR2, PDCD1LG2) concerning CAF‐related risk scores (Figure 5D) and performed Wilcoxon tests to compare checkpoint levels between CAF risk groups (Figure 5E). We found significant correlations between OLA1, CTLA4, PDCD1, CD274, IDO1, and PDCD1LG2 and the CAF risk score, with only PDCD1LG2, IDO1, and CD274 showing notably different expression levels between high‐ and low‐risk groups. This suggests a potential role of CAF‐related genes in immune suppression, aligning with existing literature. Furthermore, we observed significant differences in the proportions of Tregs, activated natural killer cells, monocytes, M0 macrophages, and eosinophils between CAF low‐ and high‐risk groups (Figure 5G). We also investigated whether Tregs and M0 macrophages were the predominant immune cell types in the TCGA‐GBM cohort (Figure 5H), analyzing their relative expression levels in the dataset (Figure S6A–D).

To provide a comprehensive assessment of the immune landscape, we employed various tools, including TIMER, CIBERSORT, CIBERSORT‐ABS, QUANTISEQ, MCPcounter, xCell, and EPIC, to analyze immune cell populations (Figure 5I). Overall, our findings highlight the relative expression of M0 macrophages and Tregs within the TCGA‐GBM dataset. Significant differences in the proportions of Tregs, activated natural killer cells, monocytes, M0 macrophages, and eosinophils were identified between CAF low‐ and high‐risk groups (Figure 5G). We assessed whether Tregs and M0 macrophages ranked as the top two immune cell types in TCGA‐GBM (Figure 5H). The relative expression levels of M0 macrophages (Figure S6A,B) and Tregs (Figure S6C,D) were analyzed in the TCGA‐GBM dataset. Furthermore, immune cell profiling was conducted using TIMER, CIBERSORT, CIBERSORT‐ABS, QUANTISEQ, MCPcounter, Xcell, and EPIC methodologies (Figure 5I). Immune function analysis revealed significant differences in several parameters, including activated dendritic cells (aDCs), antigen‐presenting cell (APC) inhibition, APC stimulation, CCRs, checkpoints, cytolytic activity, dendritic cells (DCs), macrophages, major histocompatibility complex (MHC) class I, neutrophils, parainflammation, cell inhibition, cell stimulation, T helper cells, Tregs, and type II IFN response between groups (Figure 5F). Different levels of immune cell infiltration are closely related to tumor growth and immune escape [27, 28]. The relationship between different levels of immune cell infiltration and CAF further suggests that CAF may further affect the occurrence and development of tumors by influencing the level of immune cell infiltration.

### 4.11 Sensitivity of Immunotherapy of CAF‐Risk Groups

**FIGURE 6:**
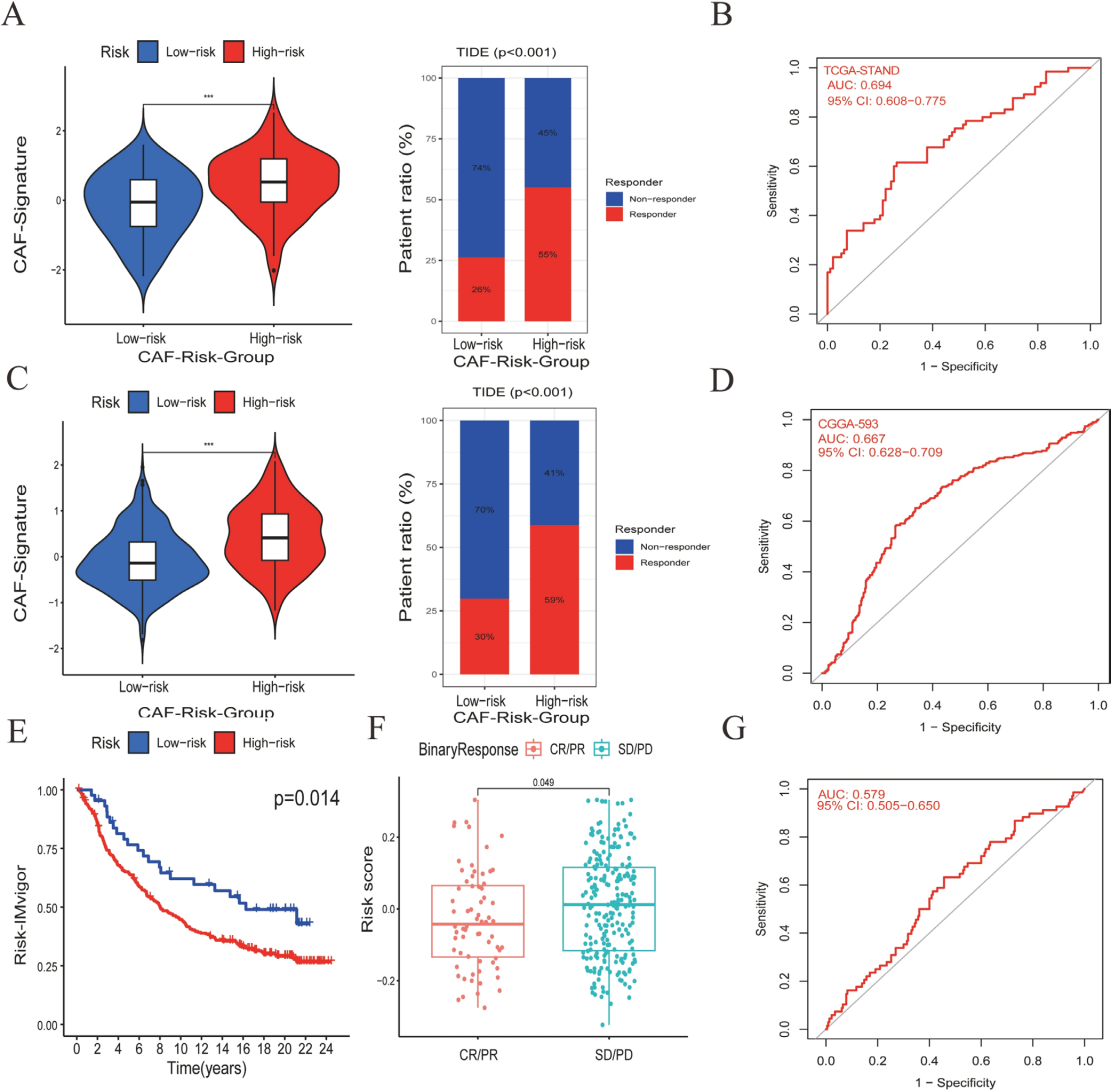
TIDE immunotherapy prediction analyses assessed the CAF risk scores of TIDE‐predicted responders and nonresponders in TCGA‐GBM (A, left) and CGGA‐PRJCA001747 (C, left). The distributions of responders and nonresponders were compared in high‐ and low‐CAF‐risk groups in TCGA‐GBM (A, right) and CGGA‐PRJCA001747 (B, right). ROC curves demonstrated the predictive capability of the CAF risk score for immunotherapy responses in TCGA‐GBM (B) and CGGA‐PRJCA001747 (D). Kaplan–Meier analyses based on the IMvigor response (E), binary response to immunotherapy (F), and the ROC curves (G) were also included. *p < 0.05, **p < 0.01, ***p < 0.001.

Immunotherapy, especially using immune checkpoint inhibitors, shows promise for patients with GBM. We employed the TIDE method to evaluate whether the CAF risk score could predict immunotherapy sensitivity in GBM patients. In the TCGA‐GBM dataset, the CAF score was significantly higher in non‐responders than in responders (Figure 6A, left). Patients with high CAF risk displayed greater immunotherapy sensitivity (55%) compared to the low CAF risk group (26%) (Figure 6A, right). Similarly, in CGGA‐PRJCA001747, non‐responders had a notably higher CAF score than responders (Figure 6C, left), with high CAF risk patients showing higher sensitivity (30%) than their low‐risk counterparts (59%) (Figure 6C, right). The AUC values of 0.694 in TCGA‐GBM (Figure 6B) and 0.667 in CGGA‐PRJCA001747 (Figure 6D) indicate the strong predictive capability of our CAF model for immunotherapy responses. Verification through IMvigor revealed that the low CAF risk group had improved overall survival compared to the high‐risk group (Figure 6E). Additionally, binary response analysis indicated that patients with stable or progressive disease had higher risk scores than those with complete or partial responses (Figure 6F), with an AUC of 0.579 (Figure 6G). These results suggest that CAF risk groups respond differently to immunotherapy.

### 4.12 Multidimensional Validation of CAF‐Risk Genes in Single‐Cell, CCLE, and HPA Databases

To validate the CAF risk genes, GSE141383 was chosen for single‐cell verification, revealing diverse cell clusters including endothelial cells, fibroblasts, malignant cells, monocytes/macrophages, and oligodendrocytes (Figure S7A,B). The Kruskal–Wallis test between different cell types showed significant differences, particularly in endothelial and fibroblast populations, across various immune and tumor microenvironment signatures (Figure S7E). Among the selected CAF risk genes, COL5A1, COL6A1, and MMP14 were predominantly expressed in fibroblasts, while ITGA5 was mainly expressed in endothelial cells, and FN1 exhibited expression in both endothelial cells and fibroblasts (Figure S7F). CCI analysis highlighted significant interactions between fibroblasts and endothelial cells (Figure S7G,H). In transcription factor (TF) analysis, RELA and AR were notably enriched in fibroblasts, whereas TAL1 was highly enriched in endothelial cells (Figure S7I,J). Additionally, the CCLE database showed higher mRNA expression levels of the five hub genes (COL5A1, COL6A1, FN1, ITGA5, and MMP14) in fibroblast cell lines than in GBM cell lines (Figure S8A,B). Protein expression analysis using IHC images from the HPA database revealed strong staining of these proteins in the GBM stroma, confirming their status as CAF‐specific markers (Figure S8C).

## 5 Discussion

GBM is a highly lethal disease, as evidenced by the fact that adults with primary GBM have a median OS of merely 1 year after diagnosis [29]. The development of GBM is critically influenced.

As CAF are major cells in the stroma, stromal scores are necessary for us to evaluate CAF in GBM. In addition, comprehensive application of MCPcounter, TIDE, xCell, and EPIC enabled us to analyze infiltration scores, immunotherapy response, immune cell subtype proportions, and CAF infiltration proportions data from two GBM cohorts, TCGA‐GBM and CGGA‐PRJCA001747 [30, 31]. We found that higher CAF and stromal scores consistently correlated with decreased OS after initial GBM treatment. This study represents the first to establish the presence of prognostic genes related to CAF in GBM. Additionally, it delineates the significance of CAF‐related genes in GBM therapy within the context of the immune environment across two distinct GBM cohorts. WGCNA is an algorithm used to generate a correlation matrix with a bicorrelation algorithm and select coexpressed genes [22]. Through the use of WGCNA, 2 gene sets were selected in TCGA‐GBM and CGGA‐PRJCA001747. Through the construction of a Venn diagram, we found 17 genes that were significantly coexpressed in both datasets. CAF are considered to mainly function in extracellular constituents, especially collagen‐containing constituents [32]. The coexpressed genes were predominantly enriched in ECM organization, along with other terms related to ECM structure and composition. Key pathways identified through KEGG analysis included focal adhesion, ECM–receptor interaction, human papillomavirus infection, and the PI3K‐Akt signaling pathway. Activation of focal adhesion signaling by CAF may contribute to CD8+ T‐cell activation inhibition and evasion from immune surveillance [33]. The PI3K‐Akt signaling pathway is recognized as a therapeutic target due to CAF involvement in various tumors [34]. Furthermore, focal adhesion, ECM‐receptor interactions, and the PI3K‐Akt signaling pathway are linked to GBM prognosis [35]. These previous findings support the enrichment results reported here, providing further evidence of the role of CAF‐related pathways in GBM prognosis.

A prognostic model for CAF was developed and validated through univariate Cox and LASSO regression analyses, incorporating five genes: COL5A1, COL6A1, FN1, ITGA5, and MMP14. In our results, the expression of five risk‐related genes was positively correlated with CAF markers. Then, five CAF genes were observed in the significant risk model based on single expression. In the analysis of chemotherapy drugs, GBM patients with elevated CAF risk scores demonstrated greater sensitivity to erlotinib and the relationship between different levels of immune cell infiltration, utilizing the median CAF risk score as the threshold. Erlotinib, an EGFR tyrosine kinase inhibitor, has been reported to interact with CAF in lung cancer therapy [33]. Simultaneously, AZD3759, an EGFR tyrosine kinase inhibitor, demonstrates enhanced blood–brain barrier penetration and has shown efficacy in managing central nervous system metastases [34]. These results suggest that EGFR may be a target of CAF in GBM therapy. In our study, we employed GSEA to explore the mechanistic pathways linked to our CAF risk model. This analysis revealed significant enrichment of various hallmark pathways in the high CAF risk group, including EMT, hypoxia, IL‐6‐JAK‐STAT3 signaling, inflammatory pathways, TNF‐α, and allograft rejection. Furthermore, IFN‐γ gene sets were prominently enriched in this group. The results from ssGSEA indicated a positive correlation between CAF risk scores and TNF‐α, inflammation, and EMT across both cohorts. Collectively, these findings suggest that TNF‐α, inflammation, and EMT are the primary hallmarks of our risk model. Notably, Zhu et al. reported that TNF‐α can induce cyclooxygenase‐2 (COX‐2) mRNA expression in CAF, enhancing the proliferative and invasive capacities of colon cancer epithelial cells [35].

Inflammation plays an important role in CAF‐related tumor promotion [36]. CAF or their precursor cells play a significant role in promoting the EMT of malignant cells [37]. These findings support our results and indicate the significance of immune status in the CAF risk group in GBM. Tumors were ranked based on their TMB, also known as the tumor mutation load [38]. Thus, TMB levels were evaluated in GBM patient CAF risk groups. PTEN, TP53, EGFR, TTN, and MUC were the top five mutated genes in our risk model, and they have been reported to play essential roles in several tumors through immune‐related processes [39]. PTEN mutations can lead to the dysregulation of the PI3K/AKT signaling pathway, promoting cell survival and proliferation [40]. In this study, we found that high‐ and low‐TMB groups show significant differences in GBM prognosis. In terms of our risk score and TMB values, there were significant differences in the different risk score groups. These findings suggest that based on our risk model, the TMB is related to the prognosis of GBM patients. CAF represent important targets for immunotherapy in tumors, and the interaction between immune cells and CAF in cancer has been shown to be important [15, 41]. From the GSEA, ssGSEA, and TMB results, we found that our CAF risk model was mainly enriched in immune‐related hallmarks. The relationship between tumors and immunity is very close. The team of Sanqi An proposed that cancer type‐specific m6A can affect immune infiltration by participating in different immune‐related pathways, thus leading to the dysregulation of the tumor microenvironment and generating tumor heterogeneity [42]. Therefore, it is necessary to elaborate on the relationship between tumors and immunity. Using immune gene sets, we enhanced our risk model through GSEA and ssGSEA. Both analyses revealed enrichment of Tregs in the GBM immune environment. CAF are considered tumor‐immune inhibitors that cannot be ignored [43]. Kinoshita et al. reported that Tregs can be reduced by CAF in adenocarcinomas [44]. Moreover, we analyzed immune infiltration in our CAF risk model. Based on the ESTIMATE score and immune score, we found significant differences between the high CAF risk and low CAF risk groups. The analysis of five single genes in the GBM immune environment showed that the proportion of Tregs was significant in GBM. The analysis of well‐known checkpoint genes in our risk model showed that PDCD1LG2, IDO1, and CD274 were related to the CAF risk model, and they were reported as immune inhibitors in a previous study [43, 45]. Subsequent analysis of immune cell types and function suggested that CAF risk genes could impact GBM prognosis via immune inhibition. In immune cell proportion analysis, we found differences in different samples. Tregs and macrophages were also major cells in our clusters. The relative genes that correlated with macrophages and Tregs are shown in Figure S6, and they may serve as target genes for CAF‐based therapy. Moreover, analysis using the TIDE algorithm revealed that elevated CAF risk scores were associated with enhanced immunotherapeutic efficacy in GBM patients. Notably, CAF inhibit the proinflammatory features of M1 macrophages, potentially influencing the tumor microenvironment [46]. Tregs have been reported to be related to the immune inhibition of CAF in tumors [41, 44, 46, 47]. In breast cancer, CAF have been shown to attract and induce Tregs. In GBM, the accumulation of Tregs has been demonstrated in the TME and peripheral blood [48]. In fact, regulatory T cells (Tregs) have been observed to be highly enriched in a variety of malignant tumors such as liver cancer and lung cancer [49, 50]. Therefore, it is currently widely believed that the enrichment of Tregs promotes tumor immune escape and progression. The enrichment of Tregs after grouping by CAF also suggests that the expression of CAF in GBM is related to the enrichment of Tregs.

CAF are a kind of mesenchymal cell [43]. Chen et al. analyzed GBM samples with mesenchymal tumor markers [51]. Thus, the GSE141383 dataset was selected for the verification of our model. In GSE14138, previous reports of CAF markers can be found. The results show that our CAF‐risk genes can be verified in GSE141383, which further supported the conclusion that COL5A1, COL6A1, FN1, ITGA5, and MMP14 are CAF‐risk genes in GBM. In additional analysis, the five genes were predominantly expressed in endothelial cells and fibroblasts and exhibited interactions with other cell types in the GSE141383 dataset. These interactions may be based on different TFs. RELA and AR are the main TFs in fibroblasts, while TAL1 is the main TF in endothelial cells. These might be the target TFs in the CAF risk model in GBM. Analysis of the CCLE database confirmed that the expression of the five identified genes was significantly elevated in fibroblast cell lines. Furthermore, examination of immunohistochemistry (IHC) images from the HPA database demonstrated heightened protein staining in the stromal regions of GBMs. These results indicate that these genes function as specific markers for CAF in GBM, supporting the validity of our model in assessing CAF infiltration levels. Notably, COL5A1 and COL6A1, among the identified markers, have been linked to CAF and are closely associated with the progression, invasion, and metastasis of breast cancer [52]. Pancreatic ductal adenocarcinoma (PDAC) cells and CAF were both found to express FN1. ITGA5, a subunit of the fibronectin (FN) receptor, is found to be overexpressed in CAF within clinical pancreatic cancer samples. This indicates a potential interaction between FN1 and ITGA5 [53]. Recent research has underscored the importance of MMP14‐expressing CAF in the progression of stage III colorectal cancer (CRC), indicating their potential as a therapeutic target for CRC treatment [54]. In GBM, COL5A1 expression correlates with diverse genetic alterations and patient survival outcomes [55]. P4HA1 regulates CD31 expression via COL6A1 during the transition of GBM stem‐like cells to tumor endothelial cells [56]. Methylation of PTPRM triggered by FN1 fosters GBM progression by activating STAT3 signaling [57]. ITGA5 is identified as a novel oncogenic biomarker in GBM and demonstrates a correlation with the immune TME [58]. TME modulates GBM stemness through the MMP14‐DLL4‐Notch3 pathway. This finding corroborates our results. It is commonly understood that cancer cells with elevated mutation levels are more recognizable by the immune system, thereby enhancing immune response and potentially improving immunotherapeutic outcomes [59]. Our analysis proposes a mechanism in which increased TMB may amplify tumor‐killing effects by affecting stromal fibrillation and weakening the local microenvironment. However, additional experiments are needed to clarify the relationship between CAF and TMB. EMT confers invasive properties to polarized epithelial cells, with TGF‐β signaling playing a role in the activation of CAF within tumors [60]. CAF can interact synergistically to initiate and enhance the process of EMT [61, 62]. CAF play a dominant role in fostering uncontrolled angiogenesis by inducing a hypoxic TME [63] and secreting proangiogenic factors like galectin‐1 [64], vascular endothelial growth factor (VEGF) [65], and hepatocyte growth factor (HGF).

Several limitations of our study should be acknowledged. In the calculation of risks associated with basic clinical features, it can be found that in TCGA‐GBM, there are statistically significant differences based on age, IDH mutation, and 1p19q codeletion status (Table 1; Figure S1A). Meanwhile, in the CGGA‐PRJCA001747, statistically significant differences exist in terms of age, WHO grade, IDH mutation, and 1p19q codeletion status (Table 2; Figure S1D). In the study of glioma, age, World Health Organization (WHO) grade, isocitrate dehydrogenase (IDH) mutation, and 1p19q codeletion status are crucial factors that affect patient prognosis and treatment options. Therefore, they are worthy of attention as clinical features of GBM. age can reflect the basic distribution state of the enrolled patients. According to the malignancy degree of glioma, glioma can be classified from benign to malignant into grades I–IV, and such grading can provide information for clinical decision‐making. The IDH mutation and 1p19q codeletion status indicate a lower malignancy of glioma. Apparently, in global databases like TCGA and CGGA, it is reasonable and acceptable that there are differences in the basic clinical features of glioma patients from around the world. In future further analysis and clinical sample collection, we should strive to expand the sample size and specify more reasonable clinical samples. Standardized basic clinical features will make the independent factors we observe more accurate and reliable. Moreover, TMB is a newly proposed concept in recent times, which specifically refers to the number of mutations present in the genome of tumor cells. However, currently, there are relatively few studies in GBM. Therefore, establishing a connection between TMB and GBM is of innovative and revelatory significance. In addition, future exploration could involve further detecting the mutated tumor genomes (PTEN, TP53, EGFR, TTN, and MUC) that are highly correlated with TMB in additional samples or inducing mutations in model cells through molecular biotechnology. This retrospective bioinformatics analysis relied on two public gene expression datasets, and the prognostic and therapeutic implications of the CAF model require validation in multicenter, prospective studies. Additionally, further investigation into the biological functions of the CAF signature biomarkers in GBM through molecular and animal experiments is essential. Nevertheless, our findings offer important insights to inform future research on the role of CAF in GBM.

In conclusion, this study is the first to provide evidence for the existence of CAF in GBM. The risk model we developed, utilizing five risk signatures (ITGA5, MMP14, FN1, COL5A1, and COL6A1) associated with CAF, holds promise for predicting GBM prognosis and guiding treatment decisions for patients. The immune status, chemotherapy, and immunotherapy responses were analyzed, and possible mechanisms for CAF in GBM were revealed. These findings underscored the potential target of CAF signatures in GBM and provide evidence for CAF‐related GBM therapy studies.

## Author Contributions

**Haifeng He:** conceptualization, methodology, software, writing – original draft preparation. **Min Yan:** data curation, writing – original draft preparation. **Ke Ye:** visualization, investigation. **Rui Shi:** supervision. **Luqing Tong:** software, validation. **Shengxiang Zhang:** writing – reviewing and editing. **Yu Zhu:** writing – reviewing and editing. **Renya Zhan:** writing – reviewing and editing.

## Ethics Statement

The authors have nothing to report.

## Conflicts of Interest

The authors declare no conflicts of interest.

## Supporting information


**Figure S1.** The clinical characteristics of TCGA‐GBM and CGGA‐PRJCA001747 cohorts. (A) Nomogram plot, (B) ROC curve, and (C) chi‐square test analysis in TCGA‐GBM; (D) nomogram plot, (E) ROC curve, and (F) chi‐square test analysis in CGGA‐PRJCA001747.

**Figure S2.** Kaplan–Meier analysis of five individual genes and their chemotherapeutic drug response. (A) Kaplan–Meier survival analysis for specific genes in the TCGA‐GBM and CGGA‐PRJCA001747 cohorts. (B) Comparative analysis of chemotherapeutic drug responses between TCGA‐GBM and CGGA‐PRJCA001747 Cohorts. ** *p* < 0.01; *** *p* < 0.001.

**Figure S3.** GSEA and ssGSEA analysis of hallmark gene sets. (A, B) GSEA of hallmark gene sets in CAF‐high and CAF‐low risk groups within TCGA‐GBM and CGGA‐PRJCA001747 cohorts. (C, D) ssGSEA reveals positive correlation between CAF risk score and enrichment scores for TNF‐α, inflammation, and epithelial–mesenchymal transition (EMT) in both TCGA‐GBM and CGGA‐PRJCA001747 cohorts.

**Figure S4.** GSEA and ssGSEA analysis of immune gene sets. (A, B) GSEA of immune gene sets in CAF‐high and CAF‐low risk groups in TCGA‐GBM and CGGA‐PRJCA001747 cohorts. (C, D) ssGSEA results indicate a positive correlation between CAF risk score and treg enrichment scores in both TCGA‐GBM and CGGA‐PRJCA001747 cohorts.

**Figure S5.** Tumor mutation burden (TMB) analysis by CAF risk group. (A, B) Oncoplots displaying the Top 20 mutated genes in high and low CAF risk groups within the TCGA‐GBM cohort. (C) Kaplan–Meier survival analysis comparing high and low TMB. (D) Kaplan–Meier survival analysis for combined high/low TMB and high/low CAF risk groups.

**Figure S6.** Potential target genes associated with macrophages and Tregs in TCGA‐GBM. (A, B) Correlation analysis of genes associated with macrophages. (C, D) Correlation analysis of genes associated with Tregs.

**Figure S7.** Single‐cell validation in GSE‐141383. (A, B) Clustering analysis in GSE‐141383. (C, D) Bar plot of the circular plot showing cell type proportions in GSE‐141383. (E) Analysis of tertiary lymphoid structures (TLSs), Including TLS‐melanoma, T‐cell‐inflamed, IFNG, checkpoint, IMPRES, IPRES, inflammatory, CTL, and T‐quiescent markers in various cell types. (F) Expression levels of five genes across different cell types. (G, H) Interaction between fibroblasts, endothelial cells, and other cells in GSE‐141383. (I, J) transcription factor enrichment in fibroblasts and endothelial cells.

**Figure S8.** Multidimensional validation of key genes using CCLE and HPA databases. (A, B) Heatmap showing mRNA expression levels of four CAF genes in fibroblasts and GBM cell lines, with comparisons made using Wilcoxon analysis. (C) Protein expression levels of ITGA5, MMP14, FN1, COL6A1, and COL5A1 in GBM samples from the human protein atlas database.

## Data Availability

The data that support the findings of this study are available in CNR2‐24‐0828 at https://pan.baidu.com/s/1zlOPMsQK5n7wNHsiMOz5nw?pwd=meys. All raw data are available in TCGA‐GBM (https://www.cancer.gov/ccg/research/genome‐sequencing/tcga) and CGGA‐PRJCA001747 (https://cgga.org.cn/).
