# Supplementary figures and images for: Predicting Prognosis and Immunotherapy Response in Glioblastoma (GBM) With a 5‐Gene CAF‐Risk Signature
Source: Cancer Rep (Hoboken). 2025 Apr 14;8(4):e70158. doi: 10.1002/cnr2.70158 (PMC11995297; doi:10.1002/cnr2.70158)

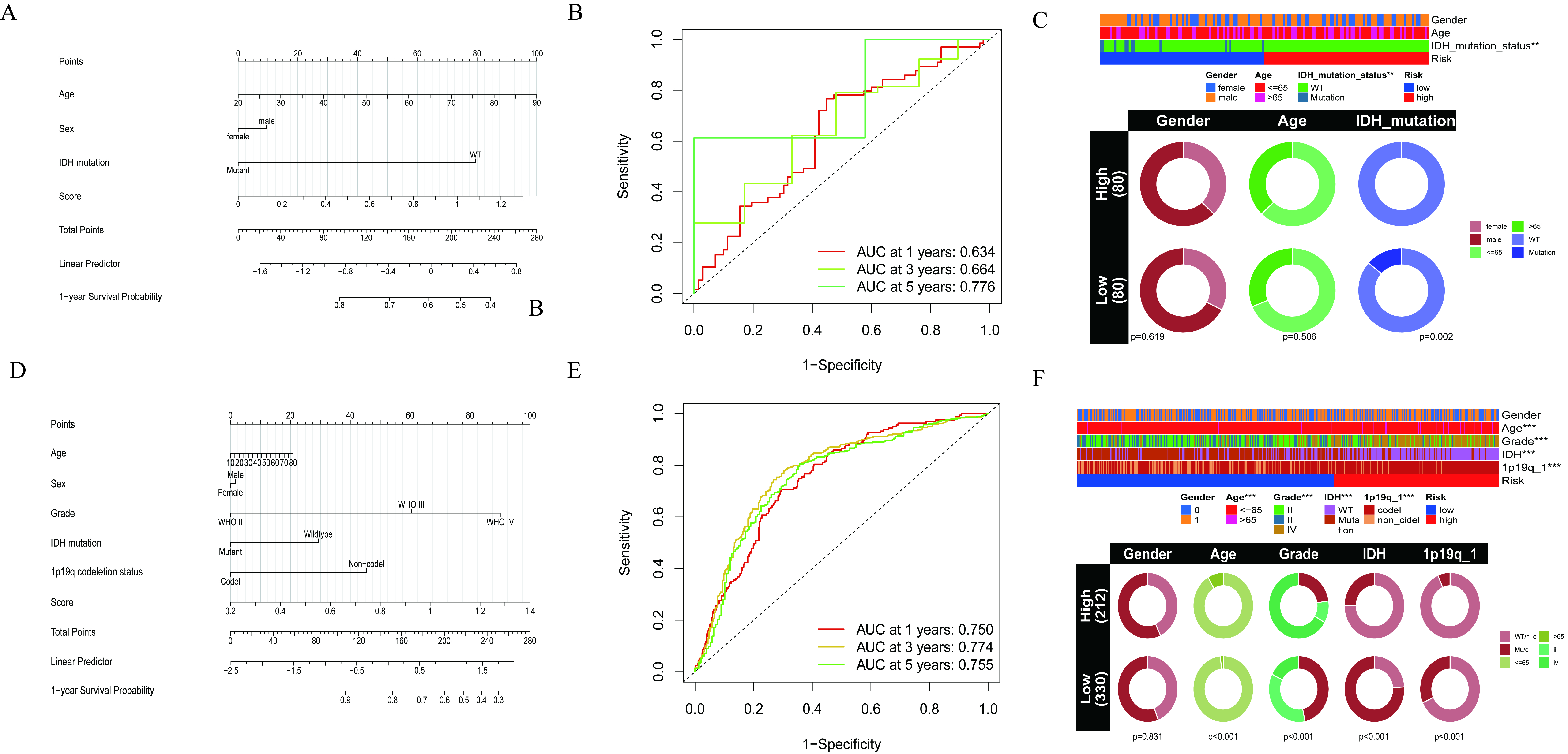

Supplement: Supplementary file 1 — Figure S1. The clinical characteristics of TCGA‐GBM and CGGA‐PRJCA001747 cohorts. (A) Nomogram plot, (B) ROC curve, and (C) chi‐square test analysis in TCGA‐GBM; (D) nomogram plot, (E) ROC curve, and (F) chi‐square test analysis in CGGA‐PRJCA001747. [file CNR2-8-e70158-s005.jpg]

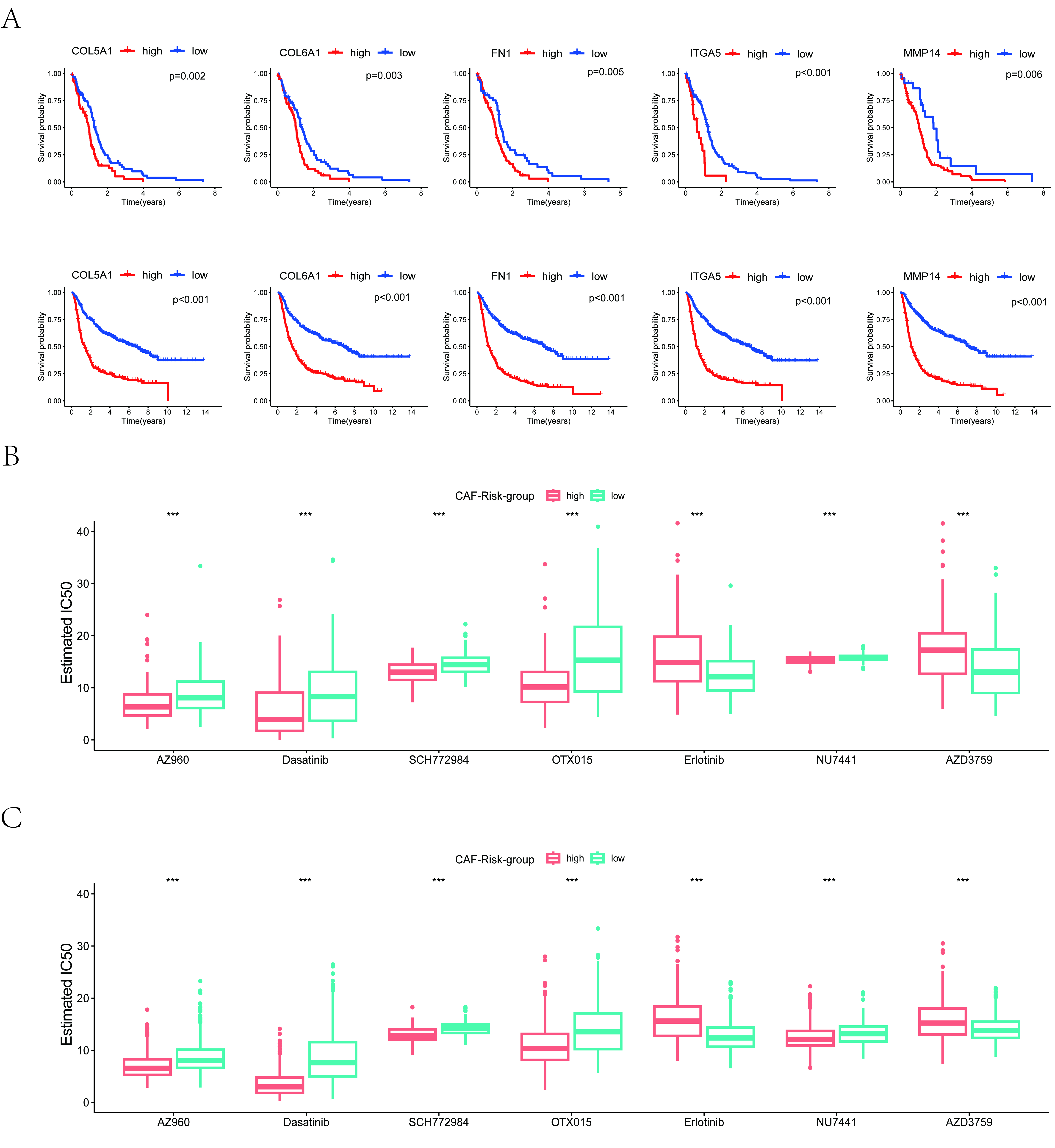

Supplement: Supplementary file 2 — Figure S2. Kaplan–Meier analysis of five individual genes and their chemotherapeutic drug response. (A) Kaplan–Meier survival analysis for specific genes in the TCGA‐GBM and CGGA‐PRJCA001747 cohorts. (B) Comparative analysis of chemotherapeutic drug responses between TCGA‐GBM and CGGA‐PRJCA001747 Cohorts. ** p < 0.01; *** p < 0.001. [file CNR2-8-e70158-s004.jpg]

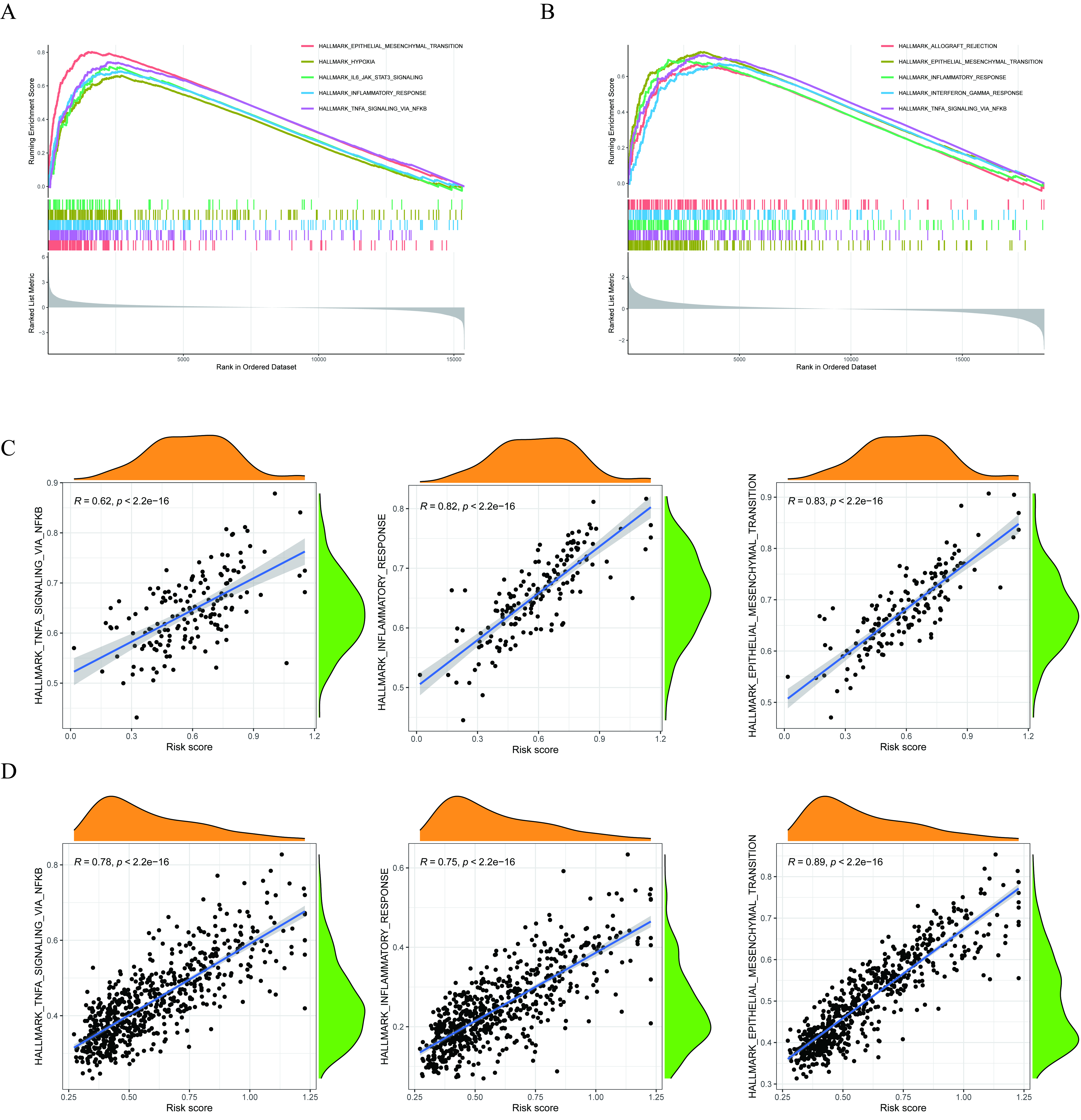

Supplement: Supplementary file 3 — Figure S3. GSEA and ssGSEA analysis of hallmark gene sets. (A, B) GSEA of hallmark gene sets in CAF‐high and CAF‐low risk groups within TCGA‐GBM and CGGA‐PRJCA001747 cohorts. (C, D) ssGSEA reveals positive correlation between CAF risk score and enrichment scores for TNF‐α, inflammation, and epithelial–mesenchymal transition (EMT) in both TCGA‐GBM and CGGA‐PRJCA001747 cohorts. [file CNR2-8-e70158-s001.jpg]

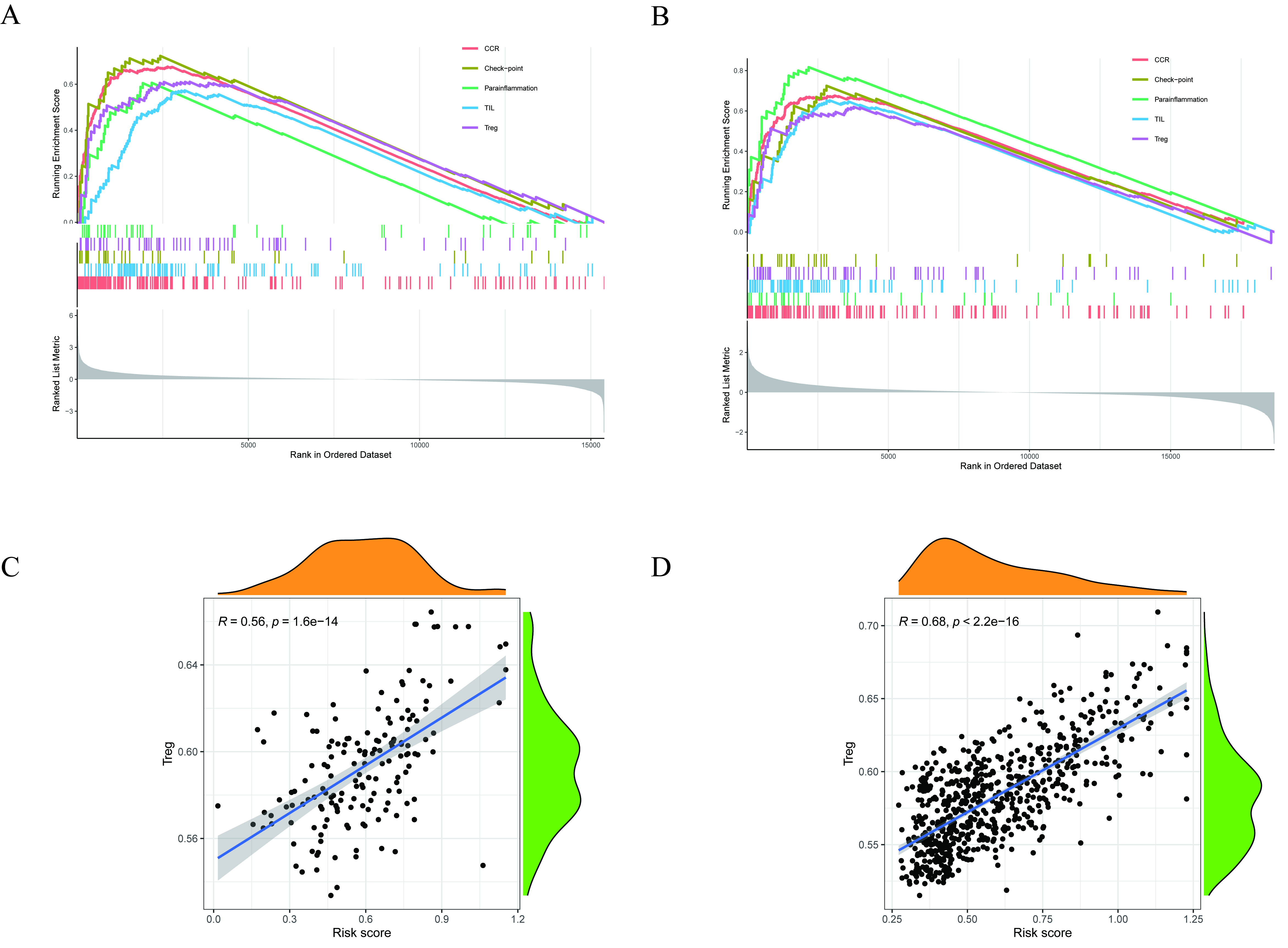

Supplement: Supplementary file 4 — Figure S4. GSEA and ssGSEA analysis of immune gene sets. (A, B) GSEA of immune gene sets in CAF‐high and CAF‐low risk groups in TCGA‐GBM and CGGA‐PRJCA001747 cohorts. (C, D) ssGSEA results indicate a positive correlation between CAF risk score and treg enrichment scores in both TCGA‐GBM and CGGA‐PRJCA001747 cohorts. [file CNR2-8-e70158-s003.jpg]

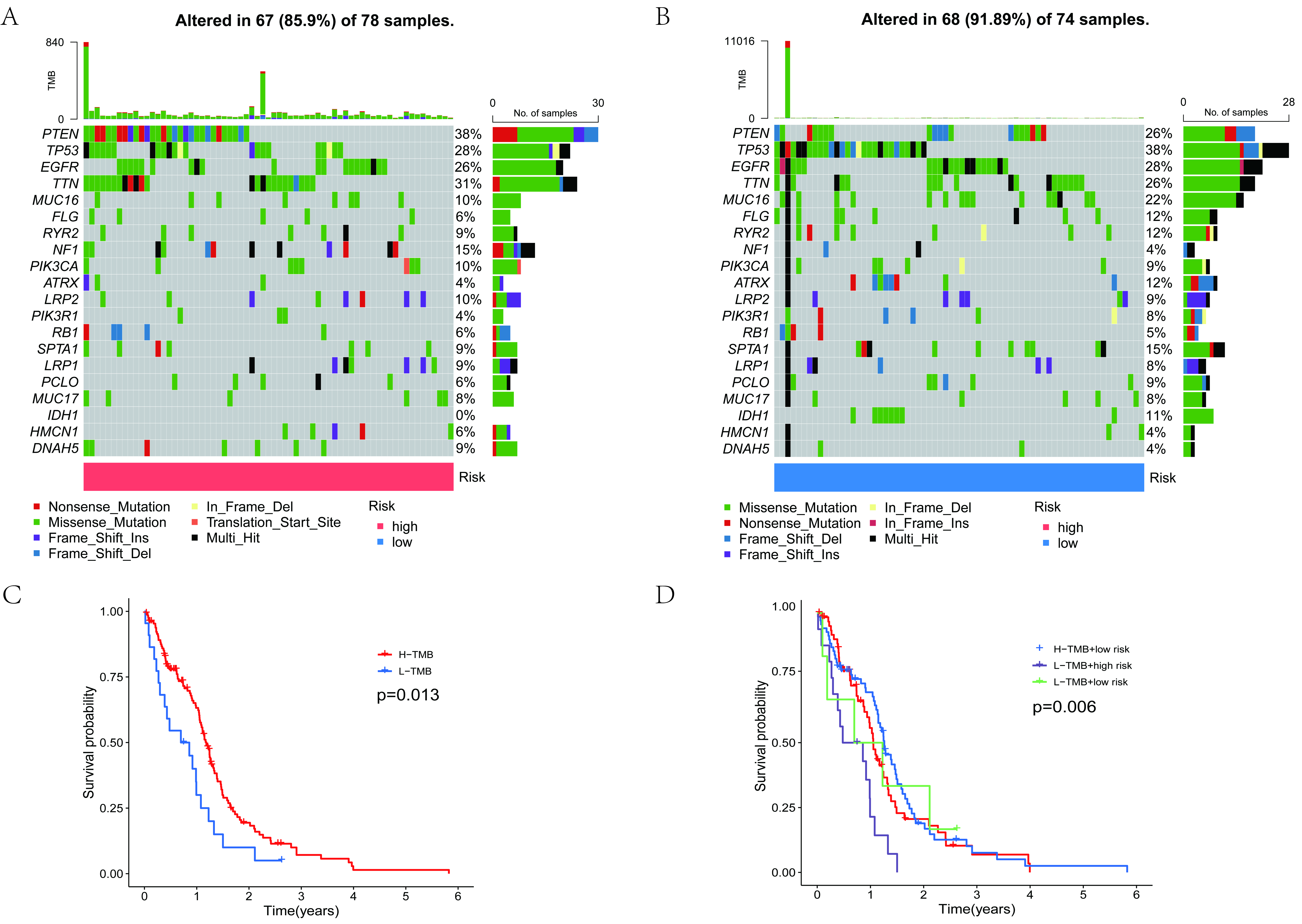

Supplement: Supplementary file 5 — Figure S5. Tumor mutation burden (TMB) analysis by CAF risk group. (A, B) Oncoplots displaying the Top 20 mutated genes in high and low CAF risk groups within the TCGA‐GBM cohort. (C) Kaplan–Meier survival analysis comparing high and low TMB. (D) Kaplan–Meier survival analysis for combined high/low TMB and high/low CAF risk groups. [file CNR2-8-e70158-s002.jpg]

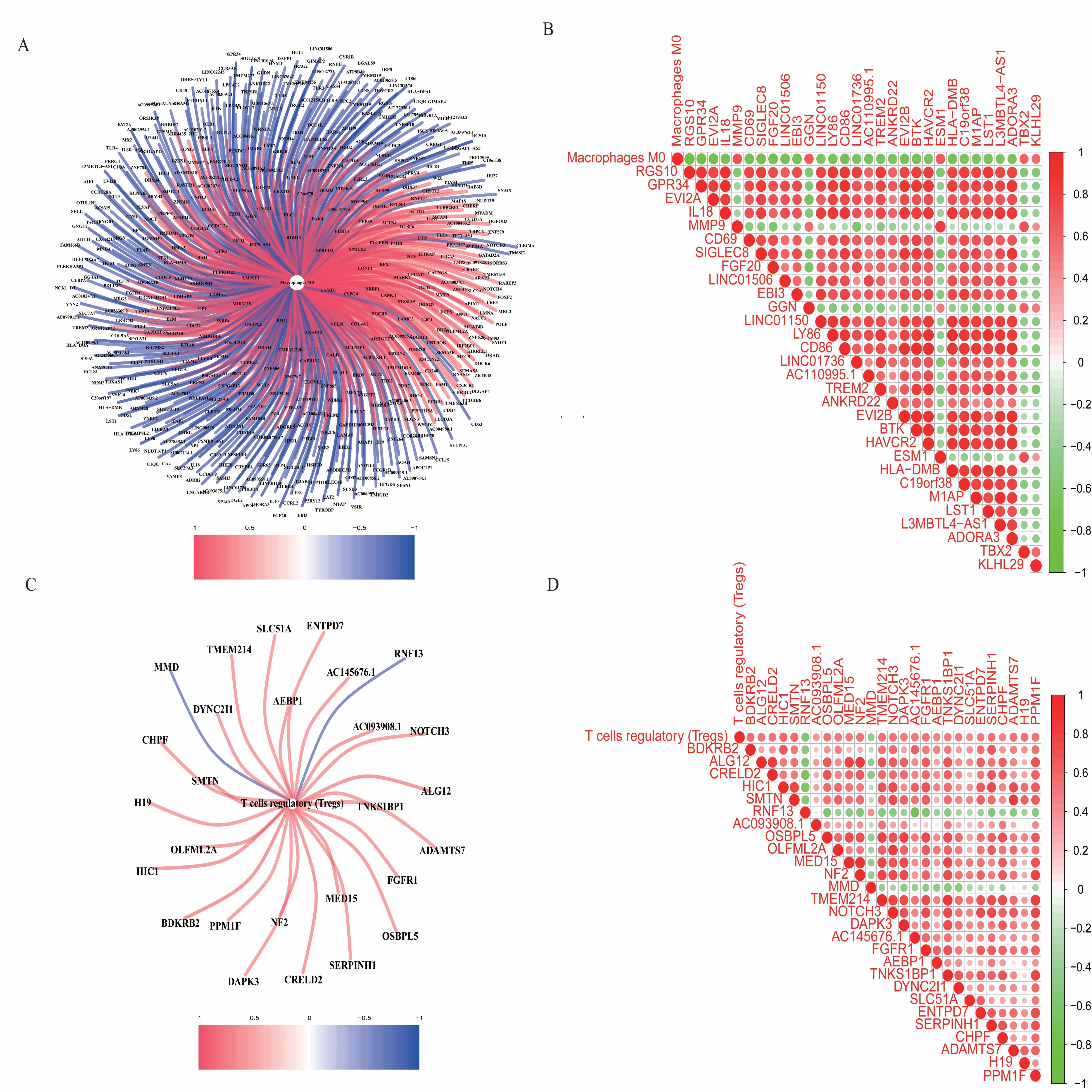

Supplement: Supplementary file 6 — Figure S6. Potential target genes associated with macrophages and Tregs in TCGA‐GBM. (A, B) Correlation analysis of genes associated with macrophages. (C, D) Correlation analysis of genes associated with Tregs. [file CNR2-8-e70158-s008.jpg]

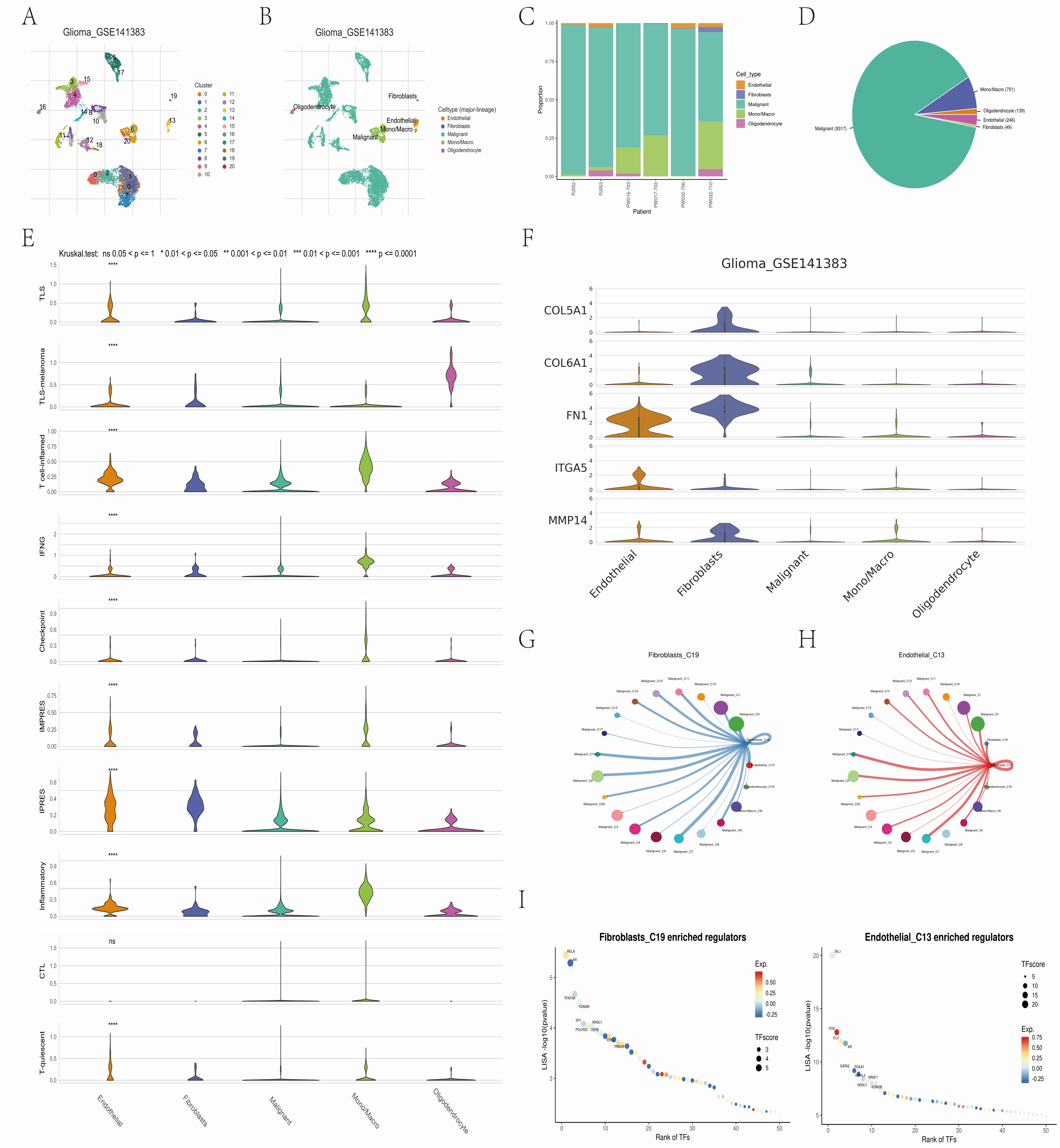

Supplement: Supplementary file 7 — Figure S7. Single‐cell validation in GSE‐141383. (A, B) Clustering analysis in GSE‐141383. (C, D) Bar plot of the circular plot showing cell type proportions in GSE‐141383. (E) Analysis of tertiary lymphoid structures (TLSs), Including TLS‐melanoma, T‐cell‐inflamed, IFNG, checkpoint, IMPRES, IPRES, inflammatory, CTL, and T‐quiescent markers in various cell types. (F) Expression levels of five genes across different cell types. (G, H) Interaction between fibroblasts, endothelial cells, and other cells in GSE‐141383. (I, J) transcription factor enrichment in fibroblasts and endothelial cells. [file CNR2-8-e70158-s006.jpg]

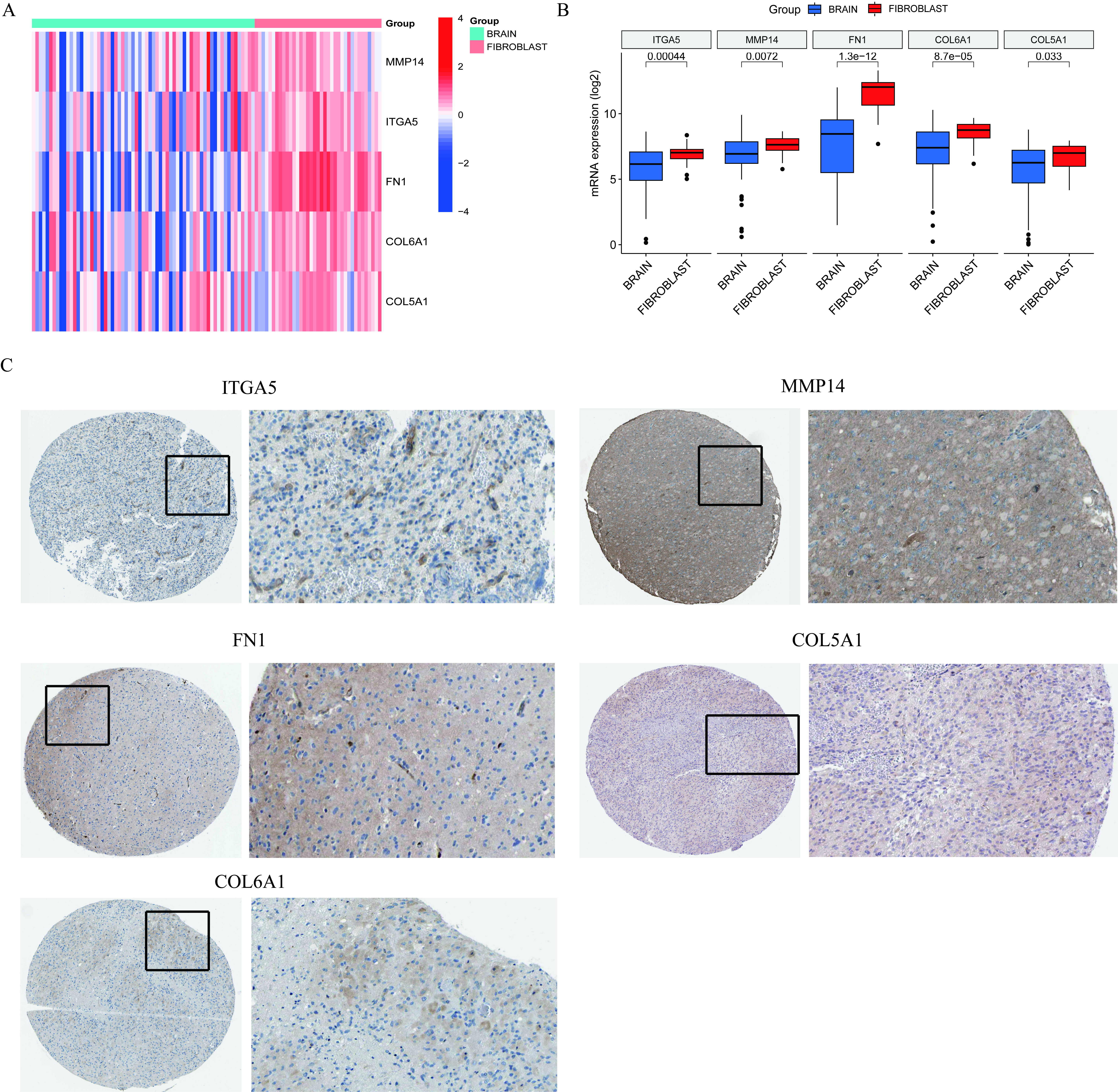

Supplement: Supplementary file 8 — Figure S8. Multidimensional validation of key genes using CCLE and HPA databases. (A, B) Heatmap showing mRNA expression levels of four CAF genes in fibroblasts and GBM cell lines, with comparisons made using Wilcoxon analysis. (C) Protein expression levels of ITGA5, MMP14, FN1, COL6A1, and COL5A1 in GBM samples from the human protein atlas database. [file CNR2-8-e70158-s007.jpg]
